# Variability of Fatty Acid Composition and Lignan Content in Sesame Germplasm, and Effect of Roasting

**DOI:** 10.1021/acsfoodscitech.3c00304

**Published:** 2023-10-02

**Authors:** Eleonora Comini, Diego Rubiales, Pierluigi Reveglia

**Affiliations:** Institute for Sustainable Agriculture, CSIC, Córdoba, 14004, Spain

**Keywords:** sesame, nutrition, unsaturated fatty acids, lignans, roasting processing, trans fatty acids, LC-MS/MS, GC-FID

## Abstract

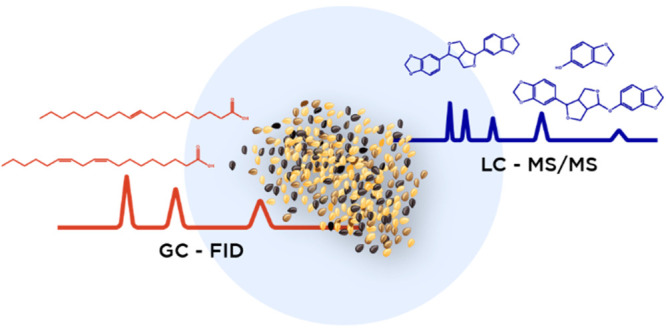

Sesame (*Sesamum indicum*) seeds are highly valued for their culinary applications and for producing a premium-quality oil. This study investigated the polyphenol content and fatty acid composition of a set of sesame accessions and examined their association with seed colors. Among the different colors, black-seeded accessions exhibited the highest total lignan content, while white-seeded accessions had average lower levels. Brown-seeded accessions showed relatively lower concentrations of sesamol and intermediate levels of sesamolin and sesamin than other colors. The oil derived from these seeds contained unsaturated fatty acids (UFAs) and saturated fatty acids (SFAs), nutritionally crucial for human consumption. Brown varieties exhibited higher concentrations of these fatty acids. Roasting black and white sesame seeds at increasing temperatures (180 and 250 °C) significantly affected lignan and UFAs concentrations. Higher temperatures resulted in elevated levels of detrimental *t*-oleic and *t*-linoleic acids. Furthermore, sesamolin content notably decreased at 180 °C and became undetectable at 250 °C. The temperature also caused a marked increase in sesamol, regardless of seed color. PCA analysis highlighted clusters between white and black varieties according to roasting temperature, displaying the potential application of chemometrics to assess processing effects and ensure sesame quality and safety. This research provides valuable insights for exploiting sesame within agrosystems in Mediterranean climates.

## Introduction

Sesame (*Sesamum indicum* L.) is one of the most ancient crops cultivated by humans. It is an important crop whose cultivation has markedly increased worldwide from 5.5 million hectares in the 1960s to the current 12.5 million hectares.^1^ Sesame seeds have many culinary applications besides providing an oil considered of high quality.^2^

The presence of lignans and great amounts of unsaturated fatty acids accounts for both the superior stability and nutritional quality of sesame oil.^3^ Lignans are secondary metabolites produced in small amounts by some plants able to exert a health-promoting action on human body.^4^ The major oil-soluble lignans in sesame are sesamin, sesamolin and sesamol.^2^ In addition to the well-demonstrated antioxidant activity,^4^ it is now recognized that polyphenols are involved in the control of aging and degenerative diseases.^5−8^ The presence of antioxidants makes sesame oil more resistant to oxidative processes, as well. Still, sesamolin is quite unstable at high temperatures, that cause its hydrolysis into sesamol, sesaminol dimer and other phenolic compounds.^9,10^

In addition to polyphenols, the fatty acid profile greatly influences the nutritional values and technological properties of sesame oil.^9^ 80% of sesame fatty acid (FA) profile is represented by unsaturated fatty acids (UFAs),^9^ with appreciable quantities of oleic acid (18:1,*n*-9) and linoleic acid (18:2,*n*-6).^2^ Sesame oil content in total monounsaturated is in line with the average of the other vegetable oils, while its content in polyunsaturated is greater than other vegetable oils, such as olive oil, canola oil and rapeseed oil.^11^ UFAs are directly associated with cardio-protective, hypolipidemic, antiatherogenic and antiinflammatory effects;^12^ conversely, high amounts of saturated fatty acids (SFAs), notably palmitic acid (C16:0) which represents the 9–12.9,%, in sesame oil causes the low-density lipoprotein (LDL) level to raise in blood, possibly resulting in cardiovascular diseases, obesity and type 2 diabetes.^13^ Nevertheless, some recent research showed that the detrimental effects of the saturated fat also depends on the macronutrient substitutions of SFAs, food matrix, the effect of the individual fatty acid and the position of the fatty acid on glycerol in triaglycerols.^14^ The FA profile affects the shelf life of sesame oil as well since oleic acid makes it far more stable to oxidation, resulting in a superior quality. Sesame seeds are commonly roasted to increase oil yield, as it enhances protein denaturation, which in turn improves lipid extractability. In addition, it enhances its sensorial properties: flavor, color, and texture, resulting in a greater consumer acceptance. However, at high temperatures the quality of oil may be reduced, possibly resulting in the formation of process-related contaminants such as polycylic aromatic hydrocarbons (PAHs).^15^

Demand for sesame products has increased in the past decade^15−18^ driven by the increasing interest of consumers in food products with high safety and quality and in tasting new ethnic products such as tahini, i.e., a 100% peeled, ground and roasted sesame paste.^19^ This reinforces the interest in expanding cultivation to other areas. Sesame has historically been cultivated mainly in tropical and subtropical areas of Africa and Asia, but its cultivation is markedly increasing in North Africa, from 0.5 million ha in the 1960s to ca. 5 million ha to date, showing the suitability of the crop to Mediterranean environments. In spite of this, cultivation is still negligible in Southern Europe with similar pedoclimatic conditions, probably due to labor costs. Sesame yields are low, with a world average of around 500 kg/ha. Breeding programs to develop adapted cultivars, with a focus on traits allowing mechanical harvest, are in progress. The nutritional profile of sesame seeds remains key. Sesame quality is greatly affected by the genotype, the environment, and the crop mangement practices. However, how these factors might interact and cause variation in sesame quality is poorly understood.^2,20−22^ In this framework, the current study aimed to evaluate fatty acid composition and lignan (sesamine, sesamolin, and sesamine) concentration in sesame germplasm grown in Southern Spain. A second objective of this study was to compare the polyphenol content and fatty acid of sesame seeds within the same color category subjected to roasting. Detailed knowledge of the agronomic features and seed composition of available sesame genotypes grown in different environments, either before or after roasting, could provide beneficial data for breeding programs for adaptability and help develop high-quality, safe products.

## Materials and Methods

### General Experimental Procedure

Distilled water, *n*-hexane and methanol were purchased from Panreac AppliChem (Barcelona, Spain). Reference standard of sesamolin was purchased from Cayman Chemical (Michigan, United States), whereas sesamin and sesamol (98%) were from Thermo Fisher Scientific (Waltham, United States). Additionally, in the experimental procedure, an electric oven (J.P. Selecta, Spain), mixer mill (MM 400 Retsch, Germany), laboratory centrifuge (DLAB, United States), Eppendorf Concentrator 26 plus/Vacufuge plus (Hamburg, Germany), and Millipore filters (13 mm, pore-size 0.20 μm, Merck, Germany) were used. The fatty acid profile analysis is carried out in a PerkinElmer Mod Clarus 500 Gas Chromatograph (Massachusetts, USA) HPLC/MS (Sciex mod 7500, QTrap)

### Plant Material

The sesame germplasm collection was grown at the experimental farm of the Institute for Sustainable Agriculture CSIC at Córdoba (Spain), between April 27 and October 20, 2022. The collection grown consisted on 510 *Sesamum indicum* accessions of worldwide origin, kindly provided by the gene banks of the Leibniz Institute of Plant Genetics and Crop Plant Research (IPK, Gatersleben, Germany), Germplasm Resources Information Network-United States Department of Agriculture (GRIN-USDA, Beltsville, M.D., U.S.A.) and other producers (Supplementary Table 1). Accessions were sown in 1 m long single rows, 0.5 m apart, 10 plants per row, with three replications using a randomized block design. The recommended agronomical and plant protection package of practices were followed for the raising of a successful crop. From germination until capsule formation, the field was sprinkler irrigated twice per week. After this, the level of irrigation was reduced to once a week. For pest control, permethrin and cypermethrin were the active ingredients selected. As for the fertilizer, 15-15-15 N-P-K was used in amounts equal to 30 kg/ha.

### Sample Selection

**Figure 1:**
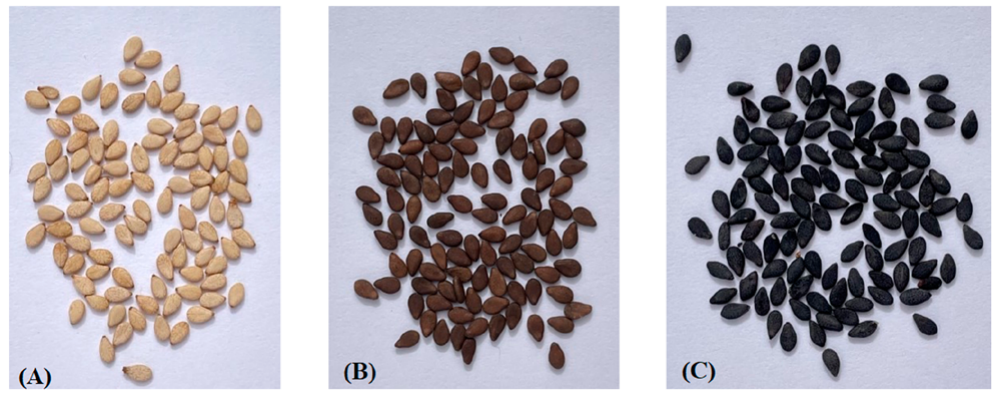
Seed coat color variation in sesame collection. (A) White (W); (B) Brown (BR); (C) Black (B).

After harvesting, the coat color of seeds was assessed. The shades ranged from black to white through all intermediate colors. To rank sesame coat colors in a semiquantitative way, RGB values, parameters which define the intensity of the color as an integer between 0 and 255, were calculated using a color capture tool (ImageColorPicker). Overall, 3 subgroups were identified: White (W), Brown (Br) and Black (B) (Figure 1). Fifty-one accessions (10% of total sowed accessions) (Table 1) were selected based on the seed availability, covering a similar proportion of accessions of all possible seed colors.

**Table 1:** List of *Sesamum indicum* Accessions Selected for Quality Analysis

Accession number	Seed coat color^a^	Accession name	Country of origin
54	W	T 59326 sel.	Texas, USA
56	W	T 60073 sel.	Texas, USA
67	W	T 61429-B-4-1-2	Texas, USA
76	W	T 62287-B-1-1	Texas, USA
79	W	T 61521-B-3-3-4	Texas, USA
87	BR	T 61502-25-1-5-1	Texas, USA
93	BR	T 61015-B-64-1-1-2	Texas, USA
110	BR	T 63039-B-3-1	Texas, USA
114	W	Hawaii-Blanco	Venezuela
118^b^	W	Nicaragua (Horvilleur)	Venezuela
123	W	7-3-5-4-2	Mexico
141	B	IP 29	India
152	B	n.a.^c^	China
153	B	n.a.	China
157	B	n.a.	China
161	B	n.a.	China
167	BR	n.a.	China
168	BR	n.a.	China
170	B	n.a.	China
173	W	Crillo	Venezuela
176	BR	43-80	Venezuela
216	B	Susam	Turkey
218	BR	1496	Turkey
224	W	1678	Turkey
228	BR	1918	Turkey
246	W	2838	Turkey
258	BR	3497	Turkey
265	BR	8669	Turkey
271	B	9797	Turkey
282^b^	B	8422	Turkey
284	B	Til	India
285	W	Til	India
292	W	Til	India
293	W	Til	India
302	B	10160	Turkey
314	BR	10222	Turkey
315	W	10361	Turkey
317	W	Til	India
318^b^	W	Til	India
324	W	Til	India
329^b^	B	Tal	India
332	W	Tal	India
358	B	11427	India
393	W	Byat-Ka-Lay	Myanmar
403	B	n.a.	India
427	BR	Konjet	Afghanistan
463	B	Taiwan White No. 2	Taiwan
473	W	INSTITUTO NO. 8	Mexico
483	BR	108	Texas, USA
484	BR	113	Texas, USA
508	B	n.a.	Thailand

a Code for color refers to Figure 1.

b Selected for processing as described in the method.

c n.a. = not available.

Moreover, 4 accessions (2 W and 2 B) were randomly selected and subjected to two treatments before extraction: (i) roasting at 180 °C for 25 min; (ii) roasting at 250 °C for 25 min.

### Sample Extraction and Preparation for Fatty Acid Analysis by GC-FID

First, 200 mg samples of selected sesame seeds (51 accessions × 3 replicates; a total of 153 samples) were dried for 24 h at 37 °C and transferred into 2 mL Eppendorf tubes. Next, each sample was milled and homogenized using a mixer mill (MM 400 Retsch, Germany) with polystyrene balls for 4 min at 30 Hz. The resulting pastes were transferred to 5 mL Eppendorf tubes. The extraction of polyphenols and fatty acids was performed as follows. Initially, 800 μL of methanol, 340 μL of water, and 800 μL of *n*-hexane (all solvents were cold) were added. The samples were vortexed for 30 s and gently stirred at 4 °C on ice for 10 min. Later, 800 μL of *n*-hexane and 800 μL of water per gram of wet tissue were added to samples. Samples were properly vortexed and centrifuged at 3000 rpm for 15 min at room temperature by a laboratory centrifuge (DLAB, United States). The upper *n*-hexane phase contains fatty acids and was collected in 1.5 mL glass vials, and the samples were dried at room temperature overnight. The samples were stored at −80 °C. Derivatization and quantification of fatty acids was performed at “Servicio Central de Apoyo a la Investigación” of Universidad de Córdoba, Spain. Briefly, fatty acids are dissolved in heptane (1 mL) and transesterified by adding 0.15 mL of KOH in methanol (10%), stirring for 5 min. Then, the phases are allowed to separate, and the heptane phase is taken for analysis by GC-FID. The fatty acid profile analysis is carried out in a PerkinElmer Mod Clarus 500 Gas Chromatograph (Massachusetts, USA), equipped with a 60-m BPX7 capillary column, 0.25 mm internal diameter, and 0.25-μm phase thickness. One μL of sample is injected under the following chromatographic conditions: injector temperature, 235 °C; column oven, 170 °C (10 min); ramp of 5 °C per minute up to 235 °C for 3 min; temperature detector FID 250 °C. The GC was used in the constant pressure mode at 25 psi of hydrogen. Identification of the methyl esters of fatty acids was done by comparing the retention times with a reference mix standard containing 55 fatty acids and taking with relative mean absolute error (MAE) errors at 1.5%. The percentage of individual fatty acid was made in relation to total area of the chromatogram (fatty acid profile), working with the area of each compound.

### Sample Preparation and Extraction for Lignan Analysis by LC-MS/MS

First, 200 mg samples of selected sesame seeds (51 accessions x 3 replicates; a total of 153 samples) were prepared and extracted as reported in the previous section. The water/methanol phases, containing the polyphenols, were transferred into 2 mL Eppendorf tubes and concentrated under vacuum by the Eppendorf Concentrator 26 plus/Vacufuge plus (Hamburg, Germany) at 30 °C. The water/methanol extracts were also filtered through Millipore filters (13 mm, pore-size 0.20 μm, Merck, Germany) into 1.5 mL glass vials prior to LC-MS analysis. Quantification of selected polyphenols (sesamin, sesamolin, and sesamol) by LC-MS/MS was performed at “Servicio Central de Apoyo a la Investigación” of Universidad de Córdoba, Spain. The samples were prepared dissolving the content of each vial into 1 mL of a mix methanol/water 50/50, and subsequently 10 mL of mobile phase were added. The resulting solutions were filtered, and 3 μL of the extract was subjected to HPLC/MS (Sciex mod 7500, QTrap). It employed a C18 column (10 cm, 0.21 mm d.u., 2.3 μm) at 40 °C with a flow of 0.35 mL/min. A calibration between 0.005 and 0.5 mg/L was made. Sample preparation was as follows: the content of each vial was dissolved in 1 mL of a 50/50 methanol/water mixture and subsequently made up to volume with 10 mL of the mobile phase. The samples were filtered and injected into HPLC/MS (Sciex mod 7500, QTrap).We worked with a C18 column (10 cm, 0.21 mm i.d., 2.3 um), flow 0.35 mL/min, column 40 °C, and 3 μl of the extract were injected.

A calibration was performed between 0.005 and 0.5 mg/L. The limit of detection (LOD) and limit of quantification (LOQ), expressed in mg/g, were equal to 0.05(LOD)/ 0.075 (LOQ), 0.05 (LOD)/0.075 (LOD) and 0.01 (LOD)/0.02 (LOQ) for sesamolin, sesamin, and sesamol, respectively.

### Statistical Data Analysis

ANOVA analysis (threshold *p* < 0.05) were carried out using SPSS (IBM version 26). Multivariate Statistical Analysis, to highlight significant variation in fatty acids and polyphenols composition, was carried out using Metaboanalyst 5.0.^23^ For multivariate statistical analysis, the data were autoscaled and transformed using logarithmic transformations.

## Results and Discussion

### Sample Harvest and Selection

After harvesting, the coat color of seeds was assessed, and the seeds were classified into 3 subgroups: White (W), Brown (Br) and Black (B) (Figure 1). Out of the full collection, 21 W, 12 B, and 14 Br were selected for analysis (Table 1).

### Nutritional Profile

**Figure 2:**
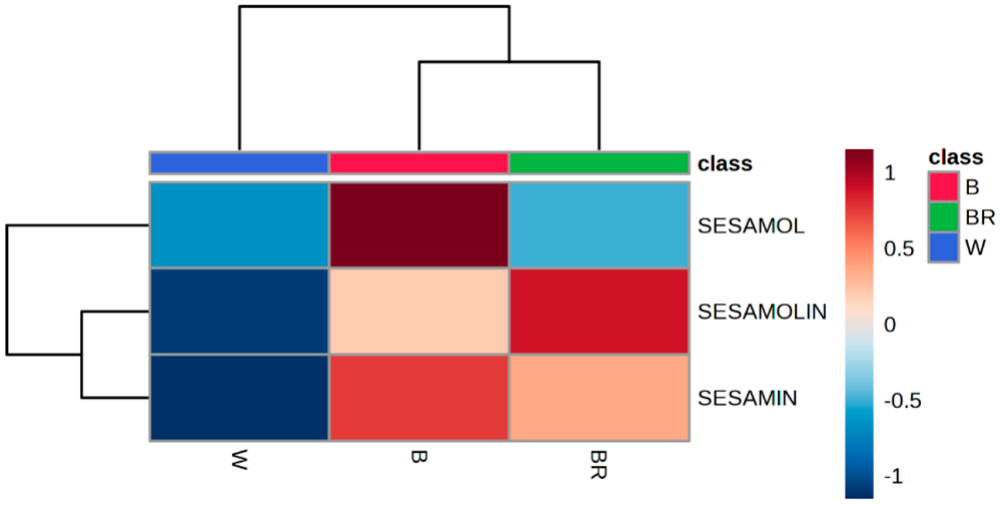
Heatmaps reporting the variation of concentration of sesamin, sesamolin and sesamol according to seed coat color.

The mean amount and range (minimum and maximum) of lignan contents for seeds color are summarized in Table 2 and illustrated in Figure 2. LC-MS/MS analysis revealed that sesamolin and sesamin are the major lignans detected in raw sesame oil, as reported in previous studies.^24,25^ The mean values of sesamin were 3.38, 4.77, and 4.61 mg/g in white, brown, and black seeded accessions, respectively. Sesamolin was also present in amounts of 2.77 2.79, and 3.19 mg/g in white, brown, and black varieties. On the other hand, sesamol, with concentrations of 0.051 mg/g, 0.059 mg/g, and 0.10 mg/g in white, brown, and black seeded accessions was the least abundant lignan (Table 2, Figure 2). Significantly, black accessions exhibited a higher content of sesamol compared to white and brown accessions (*p* = 0.002) (Table 2, Figure 2). Overall, sesame seeds with a black coat contained the highest average amount of total lignans (7.90 mg/g, Table 2), while white varieties were characterized by a lower concentration of lignans (6.21 mg/g, Table 2). Brown accessions displayed a total lignan concentration of 7.58 mg/g, resulting in an intermediate amount of sesamolin and sesamin compared to the other colors, as well as a reduced amount of sesamol. Though the role of the seed coat color is not clear in the sesame lignan biosynthesis pathway,^26^ it is reasonable to consider that black and brown sesame seeds, characterized by a darker coat, could share some characteristics with respect to the total polyphenol content. The aforementioned result is in accordance with previous studies,^2^ that draw attention to a higher concentration of sesamin and sesamolin in blacks seeds than in white and brown ones, when comparing 43 varieties of sesame from all climatic zones in India. Still, results are not in accordance with that reported by other studies,^26,27^ as the latter suggested that the lignan content was higher in white seeds than black ones. Environmental factors such as geographical region and climate, as well as variety-related differences, might be the explanation. The varieties characterized by a considerable total lignan content present a superior quality from a nutritional point of view, as these compounds exert beneficial effects to human body and make the oil less prone to oxidation; thus, a high concentration of total lignan concentration is desiderable in sesame seeds.^6,7,27^ Our study showed that the lignan contents for white, brown, and black accessions are widely variable in the studied accessions (Supplementary Tables 2–4). The sesamin content varied from 0.37 mg/g to 8.72 mg/g across the different accessions. The maximum sesamin content was observed in black seeded accessions 508, 403, and 302 (ranging from 8.72 mg/g to 8.03 mg/g), brown seeded accessions 483 and 167 (ranging from 7.54 mg/g to 7.05 mg/g), and white seeded accessions 318, 224, and 118 (ranging from 7.03 mg/g to 6.27 mg/g). In terms of sesamolin content, it ranged from 0.60 mg/g to 7.04 mg/g. The highest sesamolin content was found in brown seeded accessions 167 and 484 (ranging from 7.04 mg/g to 6.07 mg/g), black seeded accessions 508 and 161 (ranging from 6.35 mg/g to 6.28 mg/g), and white seeded accessions 332 and 67 (ranging from 5.28 mg/g to 5.20 mg/g). For sesamol, the range was from 0.01 mg/g to 0.18 mg/g. The maximum sesamol content was observed in black seeded accessions 508, 152, and 329 (ranging from 0.18 mg/g to 0.17 mg/g), brown seeded accessions 483 and 483 (0.17 mg/g, 0.13 mg/g), and white accession 473 (0.13 mg/g). Among all of the studied varieties, accession 508 exhibited the highest total lignan content of 15.25 mg/g (Supplementary Table 4).

**Table 2:** Mean Concentrations (mg/g) and Ranges of Sesamolin, Sesamin, and Sesamol and Total Lignan Content and Ranges by Sesame Seed Color

		Seed color		
Lignan concentration		White	Brown	Black
Sesamolin	Mean†	2.77 ± 1.71	2.79 ± 1.97	3.19 ± 1.71
	Minimum	0.69 ± 0.11	0.60 ± 0.13	0.72 ± 0.13
	Maximum	5.28 ± 0.91	7.04 ± 0.61	6.35 ± 0.08
Sesamin	Mean	3.38 ± 2.13	4.77 ± 1.91	4.61 ± 2.16
	Minimum	0.37 ± 0.18	1.47 ± 0.31	1.06 ± 0.08
	Maximum	7.03 ± 0.07	7.54 ± 0.62	8.72 ± 0.55
Sesamol	Mean	0.049 ± 0.033^b^	0.059 ± 0.049^b^	0.10 ± 0.045^a^
	Minimum	0.01 ± 0.01	0.01 ± 0.01	0.04 ± 0.01
	Maximum	0.13 ± 0.01	0.17 ± 0.01	0.18 ± 0.01
Total lignans	Mean	6.21 ± 2.98	7.58 ± 3.32	7.90 ± 3.30
	Minimum	1.42	3.58	2.27
	Maximum	11.58	14.18	15.25

† Mean ± SD; Means with different superscript are different (*P* < 0.05).

**Figure 3:**
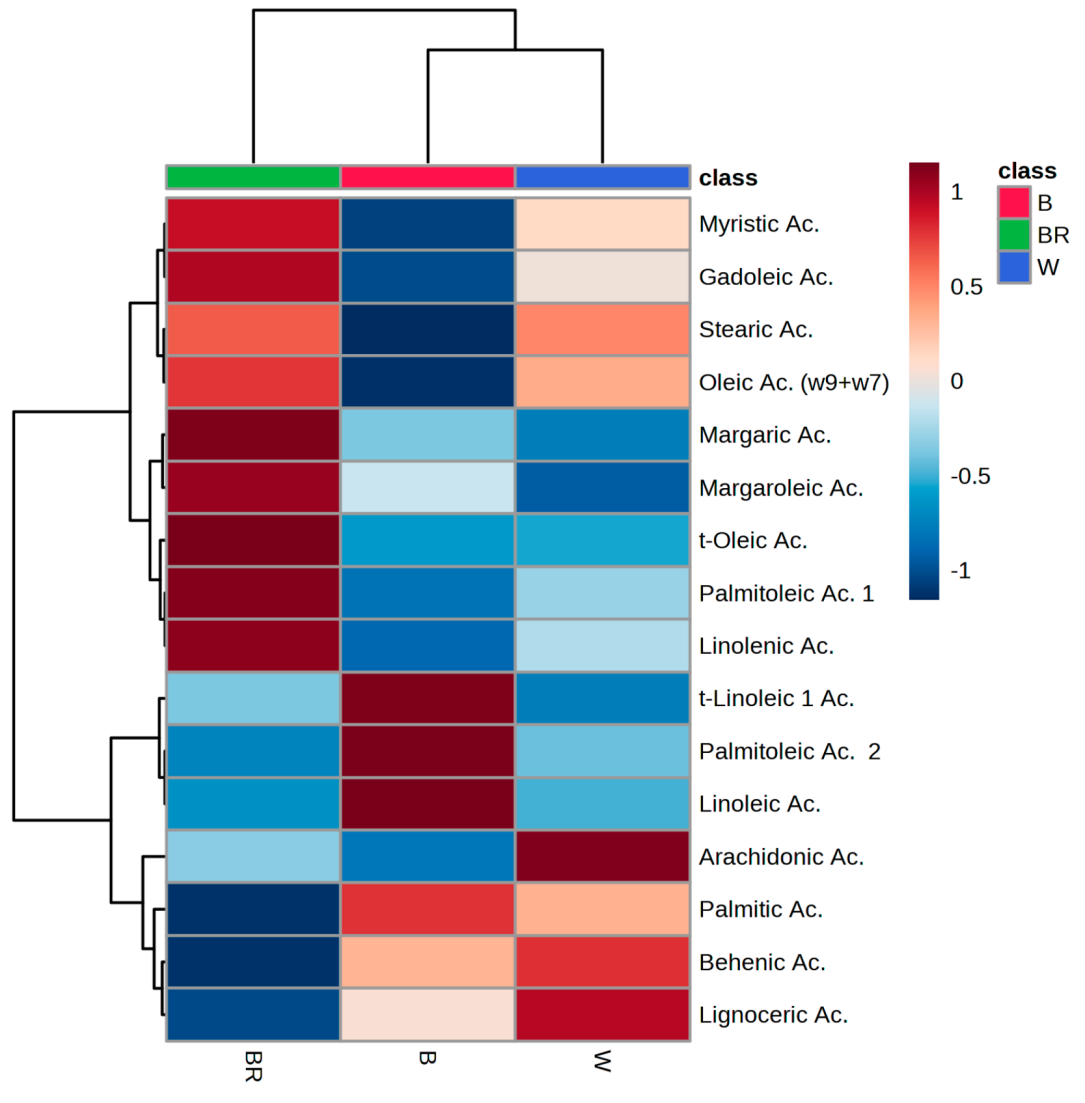
Heatmaps reporting the variation of concentration of fatty acids according to seed coat color. B = black; BR= brown; W = white.

The oil content of raw sesame seeds contained 16 identifiable fatty acids. The overall mean FAs abundances, arranged by seed color, are reported in Table 3 and visualized in the heatmap in Figure 3. Six SFAs were detected: myristic acid (C14:0), palmitic acid (C16:0), margaric acid (C17:0), stearic acid (C18:0), bahemic acid (C22:0) and lignoceric acid (C24:0), while nine UFAs were identified and quantified: palmitoleic acid (16:1), margaroleic acid (C17:1), gadolinic acid (20:1), oleic acid (C18, ω9 + ω7), *trans*-oleic acid, linoleic acid (C18:2), *trans*-linoleic acid, linolenic acid (C18:3) and arachidonic acid (C20:4). According to previous studies,^2,27^ sesame oil reported a considerable amount of UFAs, equal to 83.9% of the total fats. Within UFA, 44.6% are PUFAs, while the rest are MUFAs. Overall, UFAs in sesame oil are almost entirely represented by oleic acid (ω-7+ ω-9) and linoleic acid. Low amounts of *t*-oleic acid (0.02% g/100 g) and *t*-linoleic acid (0.04–0.06% g/100 g) were detected in all the analyzed oils. Whereas, palmitic and stearic acids were the dominant fatty acids among the SFAs, accounting for about 15.1% of the total composition (Table 3).

**Table 3:** Mean Concentrations (% g/100 g of Oil) and Ranges of FAs by Sesame Seed Color

		Seed color		
SFAs concentration		White	Brown	Black
Myristic Ac.	Mean†	0.025 ± 0.003	0.03 ± 0.003	0.02 ± 0.002
	Minimum	0.02 ± 0.001	0.02 ± 0.001	0.02 ± 0.001
	Maximum	0.03 ± 0.002	0.03 ± 0.002	0.03 ± 0.002
Palmitic Ac.	Mean	9.42 ± 0.78	9.14 ± 0.85	9.34 ± 0.74
	Minimum	8.13 ± 0.19	7.53 ± 0.05	8.54 ± 0.49
	Maximum	10.70 ± 0.14	9.50 ± 0.42	10.82 ± 0.91
Margaric Ac.	Mean	0.10 ± 0.02	0.11 ± 0.02	0.10 ± 0.01
	Minimum	0.06 ± 0.001	0.09 ± 0.01	0.08 ± 0.002
	Maximum	0.12 ± 0.01	0.14 ± 0.03	0.12 ± 0.03
Stearic Ac.	Mean	5.83 ± 0.66	5.85 ± 0.63	5.70 ± 0.69
	Minimum	4.80 ± 0.42	5.18 ± 0.37	4.96 ± 0.08
	Maximum	7.16 ± 0.53	6.69 ± 0.14	6.67 ± 0.50
Bahemic Ac.	Mean	0.13 ± 0.01	0.12 ± 0.01	0.13 ± 0.01
	Minimum	0.11 ± 0.001	0.11 ± 0.01	0.12 ± 0.004
	Maximum	0.15 ± 0.001	0.15 ± 0.01	0.16 ± 0.01
Lignoceric Ac.	Mean	0.09 ± 0.01	0.08 ± 0.01	0.09 ± 0.01
	Minimum	0.08 ± 0.01	0.08 ± 0.003	0.08 ± 0.01
	Maximum	0.10 ± 0.002	0.11 ± 0.02	0.11 ± 0.01

UFAs Concentration		White	Brown	Black
Palmitoleic Ac. 1	Mean	0.04 ± 0.01	0.045 ± 0.01	0.035 ± 0.01
	Minimum	0.03 ± 0.01	0.03 ± 0.002	0.03 ± 0.001
	Maximum	0.05 ± 0.01	0.06 ± 0.01	0.04 ± 0.01
Palmitoleic Ac. 2	Mean	0.12 ± 0.02	0.12 ± 0.02	0.13 ± 0.02
	Minimum	0.08 ± 0.002	0.08 ± 0.01	0.11 ± 0.01
	Maximum	0.15 ± 0.01	0.16 ± 0.01	0.15 ± 0.02
Margaroleic Ac.	Mean	0.05 ± 0.01	0.06 ± 0.01	0.05 ± 0.01
	Minimum	0.03 ± 0.001	0.04 ± 0.001	0.04 ± 0.004
	Maximum	0.06 ± 0.001	0.08 ± 0.02	0.07 ± 0.02
Gadoleic Ac.	Mean	0.21 ± 0.02	0.25 ± 0.01	0.19 ± 0.01
	Minimum	0.15 ± 0.03	0.16 ± 0.01	0.16 ± 0.01
	Maximum	0.21 ± 0.01	0.26 ± 0.01	0.21 ± 0.02
Oleic Ac. (ω9 + ω7)	Mean	46.53 ± 3.67	46.87 ± 3.88^a^	45.22 ± 3.81^b^
	Minimum	42.19 ± 1.28	42.64 ± 3.05	40.35 ± 2.34
	Maximum	52.75 ± 1.00	51.71 ± 0.50	51.05 ± 3.40
*t*-Oleic Ac.	Mean	0.02 ± 0.002	0.022 ± 0.002	0.02 ± 0.002
	Minimum	0.02 ± 0.001	0.02 ± 0.001	0.02 ± 0.001
	Maximum	0.02 ± 0.015	0.025 ± 0.004	0.02 ± 0.004
Linoleic Ac.	Mean	36.47 ± 3.75^b^	36.38 ± 3.90^b^	37.97 ± 3.80^a^
	Minimum	30.48 ± 1.28	32.17 ± 0.44	32.57 ± 3.51
	Maximum	40.11 ± 1.27	41.08 ± 3.09	42.27 ± 2.24
*t*-Linoleic Ac. 1	Mean	0.035 ± 0.004	0.04 ± 0.005	0.045 ± 0.005
	Minimum	0.03 ± 0.002	0.03 ± 0.002	0.03 ± 0.001
	Maximum	0.04 ± 0.004	0.05 ± 0.003	0.06 ± 0.01
Linolenic Ac.	Mean	0.30 ± 0.06	0.32 ± 0.05^a^	0.29 ± 0.06^b^
	Minimum	0.23 ± 0.01	0.27 ± 0.02	0.22 ± 0.01
	Maximum	0.48 ± 0.09	0.41 ± 0.08	0.42 ± 0.12
Arachidonic Ac.	Mean	0.64 ± 0.06	0.62 ± 0.06	0.62 ± 0.06
	Minimum	0.56 ± 0.02	0.55 ± 0.04	0.56 ± 0.03
	Maximum	0.76 ± 0.04	0.72 ± 0.04	0.69 ± 0.01

† Mean ± SD; Means with different superscript are different (*P* < 0.05).

Present findings suggests that FA profile changes depending on the color of sesame. Brown varieties contained higher concentrations of several fatty acids (i.e., miristic acid, gadolenic acid, oleic acid (ω-7+ ω-9), margaric acid, margaroleic acid, *t*-oleic acid, palmitoleic 1 acid, linolenic acid) compared to the other colors (Table 3, Figure 3). In particular, brown sesame contained statistically significant higher amounts of oleic acid (ω-7+ ω-9) compared to black (*p* = 0.037) varieties, and of linolenic acid compared to black (*p* = 0.015) varieties. The presence of oleic acid makes sesame oil beneficial to human health, since its intake has been linked to reduction of food intake and lower of glucose production, lowering of LDL cholesterol, decreased metabolic dysfunction and protection against cardiovascular insulin resistance, mainly.^28,29^ However, the evidence showing a greater amount of oleic acid in brown varieties results to be in contrast with what suggested by Dar and coauthors,^2^ who pointed out a greater concentration of oleic acid in black sesame seeds than white and brown seeds. The environmental factors like climate and location might be a possible reason for these variations, as in the aforementioned study, plants were cultivated in India. Additionally, the difference in genetic makeup could be a contributing factor as well. Moreover, statistical analysis also suggested that the black variety contains the greatest amount of linoleic acid than brown accesions (*p* = 0.044) and white accessions (*p* = 0.046), and it is low in palmitoleic 1 acid, whose effect is still controversial (Table 3, Figure 3).^30^ Conversely, palmitic acid, behenic acid, and lignoceric acid are present in slightly higher amounts in white and black sesame (Table 3, Figure 3). A higher concentration of very long-saturated fatty acids makes sesame seeds nutritionally important for the human diet, as they are associated with healthy blood lipid levels. Consumption of large amounts of the aforementioned fatty acids is linked to a lower risk of developing type 2 diabetes.^31,32^

Very recently, WHO Guidelines (2023), suggested to replace animal fats with oils rich in polyunsaturated fats, such as soybean, canola (rapeseed), corn, safflower and sunflower oils, to reduce saturated fat and industrially produced *trans-*fat intake.^33,34^ PUFA levels in corn and sunflower oils, for instance, account for 57.2 and 60.6, %, while sesame oil accounts for only 43.5%.^35^ Consequently, breeding programs aimed to increase the PUFAs content in sesame would greatly enhance sesame oil nutritional quality.

Considering the nutritional importance of oleic acid and linoleic acid, the most abundant FAs in sesame oil, the next step concerned the possibility of specific genotypes containing notably higher amounts of these UFAs. ANOVA analysis using genotypes as factors was performed. The FAs abundances in all the studied varieties, expressed in % (g/100 g of oil), are listed in Supplementary Tables 5–7, respectively. In accessions producing white sesame seeds, the richest in oleic acid (ω-7+ ω-9) were accessions 67, 79, and 473 (51.42%, 50.60%, and 52.75%, respectively), and the amounts were stastistically significant compared to accessions 56, 114, 224, 284, 292, 293, 315, 317, 324, and 393 (Supplementary Table 5). Moreover, genotype 473, from Mexico, showed a higher of oleic acid (ω-7 + ω-9) amount than 54, 76, 118, 315, 318, and 332 (Supplementary Table 5). In accessions producing brown sesame seeds, the richest in oleic acid (ω-7+ ω-9) were accessions 93, 110, and 483 (49.96, 51.71 and 50.52%, respectively) coming from the United States. Moreover, the amount of oleic acid (ω-7+ ω-9) was statistically significant compared to accessions 168, 218, 228, and 258 (Supplementary Table 6), originating from other countries. Finally, accession 110, from the United States, showed a higher amount than 265 and 318, from Turkey (Supplementary Table 6). Among the genotypes producing black seeds, accession 216, originating from Turkey, together with sesame 329 and sesame 403, originating from India, showed the highest average concentration of linoleic acid (42.14%, 42.27% and 42.70%, respectively). While accession number 157, originating from China, showed the highest content of oleic acid (ω-7+ ω-9) followed by accessions 508 and 282 (51.05%, 49.02% and 48.71%, respectively) (Supplementary Table 7).

Taking into account total lignan, oleic acid, and linoleic acid content, accession 67 produces white seeds originating from Texas; accession 483 produces brown seeds also originating from Texas; and accesions 403 and 508 produce black seeds are promising candidates for use in breeding programs for further investigating how genetic and environmental factors influence sesame seeds’ quality. Comprehending the genetic mechanisms underlying the variation in these physiologically active compounds is crucial. Additionally, exploring potential links between the two biosynthetic pathways and understanding the impact of the environment on these compounds is essential for enhancing the qualitative and quantitative traits of sesame.

### Effect of Roasting on Nutritional Profile

The lignan content in raw and roasted sesame seeds was explored, as roasting is commonly applied to sesame seeds to increase oil yield and improve sensorial properties.^15,25^ Selected white and black seeded accessions. The lignan levels in white accessions 118 and 318, and black ones 282 and 329 before and after roasting are reported in Table 4. The thermal treatments in both white and black accessions heavily affect sesamolin, sesamin and sesamol content. Indeed, sesamolin was not detected after roasting at 250 °C for 25 min. The absence of sesamolin in these samples could be explained by the heating process promoting its conversion to sesamol, sesaminol dimer and other lignans.^3^ Likewise, sesamin content decreases in either white or black color-seeded varieties when roasting is applied, still the difference is not significant. Sesamol concentration increased when thermal treatment in an amount proportional to the temperature is applied: the higher the temperature, the higher the concentration. ANOVA statistical analysis confirmed that the abundance of sesamol is more remarkable in white sesame seeds roasted at 180 and 250 °C (*p* < 0.001) than white raw sesame seeds. Moreover, statistical analyses confirmed that the concentration of sesamol in black sesame seeds roasted at 180 and 250 °C (*p* < 0.001) was greater than the raw black seeds. Nevertheless, in the case of black seeds, the impact of selected temperatures on increasing sesamol concentration was lower than on white seeds. The findings are consistent with previous studies^9,10,25^ that report the conversion of sesamolin to sesamol, as discussed above. Furthermore, a recent study reports that at higher temperatures, sesamol dimerizes into sesamol dimer, which possesses antioxidant activity, but is still in a meagre amount in refined oil.^25^

**Table 4:** Effect of Roasting on the Content (mg/g) of Sesamolin, Sesamin, and Sesamol in Seeds of Selected Accessions of Sesame†

Seeds color	Temperature	Variety	Sesamolin	Sesamin	Sesamol
White	not roasted	118	3.15 ± 0.70	6.27 ± 1.33	0.08 ± 0.01^c^
	180 °C	118	3.12 ± 0.60	5.60 ± 0.33	2.62 ± 0.74^b^
	250 °C	118	n.d.	3.80 ± 1.30	8.07 ± 0.92^a^
	not roasted	318	2.92 ± 0.45	7.03 ± 0.07	0.02 ± 0.00^c^
	180 °C	318	2.30 ± 0.22	6.51 ± 0.41	0.97 ± 0.13^b^
	250 °C	318	n.d.	6.31 ± 0.45	4.98 ± 0.31^a^
Black	not roasted	282	0.72 ± 0.13	2.45 ± 1.73	0.08 ± 0.03^b^
	180 °C	282	0.35 ± 0.08	0.27 ± 0.02	4.23 ± 1.00^a^
	250 °C	282	n.d.	0.31 ± 0.06	4.56 ± 0.38^a^
	not roasted	329	2.80 ± 0.03	5.31 ± 3.40	0.17 ± 0.01^b^
	180 °C	329	2.71 ± 0.24	3.65 ± 0.41	1.80 ± 0.32^a^
	250 °C	329	n.d.	3.17 ± 1.46	2.06 ± 0.62^a^

† Mean ± SD; Means with different superscript are different (*P* < 0.05).

The oil content of roasted sesame seeds contained 18 identifiable FAs. The complete results on the effect of roasting processing on the fatty acid composition are reported in Supplementary Table 8, while the most affected FAs by the thermal treatment are reported in Table 5. As a general result, the roasting process has an impact on the FAs composition as it increases the content in UFAs, in the *trans* configuration mainly. As a consequence, the nutritional quality of derived sesame oil is affected. The present findings are in accordance with the previous studies conducted by Arab and coauthor.^36^ The roasting process has a strong effect on *t*-oleic acid. Its % (g/100 g of oil) increased with the temperature, and the differences with the raw seeds is statistically significant (*p* < 0.01), regardless of the color. The roasting process, at 250 °C, caused a statistically significant rise of the % (g/100 g of oil) of *t*-linoleic 1 acid in both white and black seeds (*p* < 0.01). Moreover, *t*-linoleic acid 2 was detected in both white and black seeds only after the roasting process, regardless of temperature (Table 3), and its % increased with the temperature. *t*-Linolenic acid was only detected when the selected seeds were heated at 250 °C for 25 min. Finally, the linolenic acid concentration decreased in white and black seeds when treated at 250 °C for 25 min.

**Table 5:** Fatty Acids Affected by Roasting Process, Composition Expressed in % (g/100 g of Oil)

	*t*-Oleic acid†	*t*-Linoleic 1 acid	*t*-Linoleic 2 acid	*t*-Linolenic acid	Linolenic acid
White					
118 (n.r)	0.02 ± 0.005^b^	0.04 ± 0.01^b^	n.d.	n.d.	0.30 ± 0.01^a^
118 (180 °C)	0.11 ± 0.03^b^	0.10 ± 0.003^b^	0.05 ± 0.01	n.d.	0.29 ± 0.01^b^
118 (250 °C)	1.11 ± 0.12^a^	0.90 ± 0.09^a^	0.83 ± 0.08	0.01 ± 0.002	0.24 ± 0.01^b^
318 (n.r)	0.02 ± 0.001^c^	0.04 ± 0.004^b^	n.d.	n.d.	0.23 ± 0.01^a^
318 (180 °C)	0.12 ± 0.02^b^	0.11 ± 0.01^b^	0.06 ± 0.01	n.d.	0.23 ± 0.01^ab^
318 (250 °C)	1.12 ± 0.06^a^	0.95 ± 0.06^a^	0.88 ± 0.05	0.01 ± 0.004	0.19 ± 0.01^b^
Black					
282 (n.r.)	0.02 ± 0.004^b^	0.03 ± 0.01^b^	n.d.	n.d.	0.26 ± 0.04
282 (180 °C)	0.15 ± 0.01^b^	0.12 ± 0.01^b^	0.07 ± 0.01	n.d.	0.28 ± 0.01
282 (250 °C)	1.29 ± 0.11^a^	0.88 ± 0.11^a^	0.82 ± 0.10	0.01 ± 0.002	0.23 ± 0.01
329 (n.r)	0.02 ± 0.004^b^	0.05 ± 0.01^c^	n.d.	n.d.	0.26 ± 0.01^a^
329 (180 °C)	0.10 ± 0.02^b^	0.11 ± 0.02^b^	0.06 ± 0.02	n.d.	0.27 ± 0.01^a^
329 (250 °C)	0.99 ± 0.13^a^	0.99 ± 0.04^a^	0.91 ± 0.05	0.01 ± 0.003	0.22 ± 0.01^b^

† Mean ± SD; Means with different superscript are different (*P* < 0.05).

**Figure 4:**
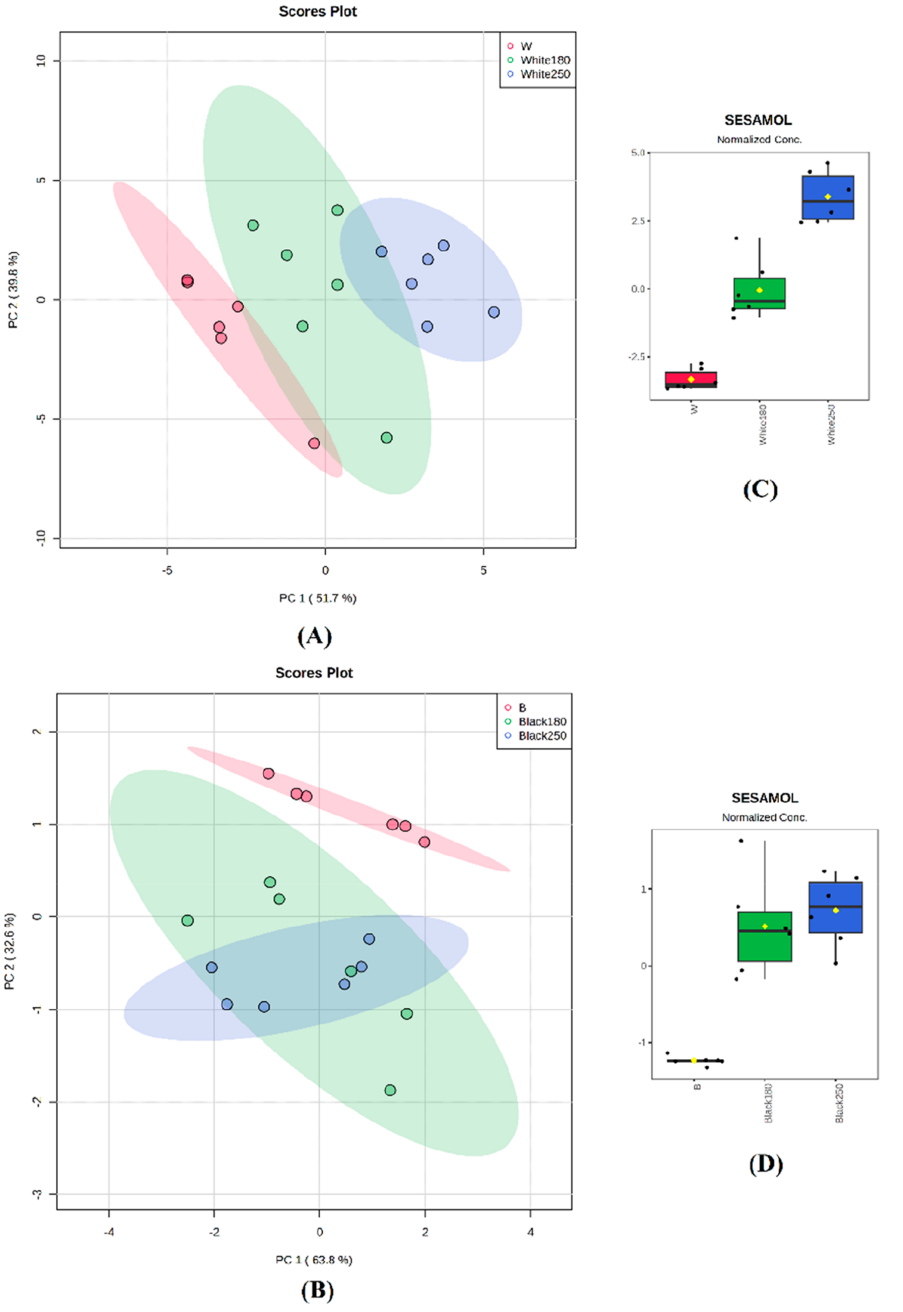
PCA analysis of selected sesame variety subjected to roasting process using polyphenols concentrations. (A) Score plot for white (W) coat color seeds; (B) score plot for black (B) coat color seeds; (C) box plots reporting the variation of sesamol concentrations in the three different treatments for white coat color seeds: not roasted (red), roasted at 180 °C (green), roasted at 250 °C (blue); (D) box plots reporting the variation of sesamol concentrations in the three different treatments for black coat color seeds: not roasted (red), roasted at 180 °C (green), roasted at 250 °C (blue).

In order to identify underlying patterns and relationships within the FAs and polyphenol composition and the roasting process, the data were also submitted to multivariate statistical analysis. In detail, data were explored by using principal component analysis (PCA) to identify the most important variables and potentially identify clusters. Figure 4 reports the PCA score plots for white (Figure 4A) and black (Figure 4B) sesame seeds. For white seeds, the score plot indicated that PC1 and PC2 accounted for 91.5% of the total variability and that three different clusters could be detected according to the thermal treatment. The most important variable accounting for the clustering was sesamol (Figure 4C). For black seeds, the score plot indicated that PC1 and PC2 accounted for 96.4% of the total variability.

**Figure 5:**
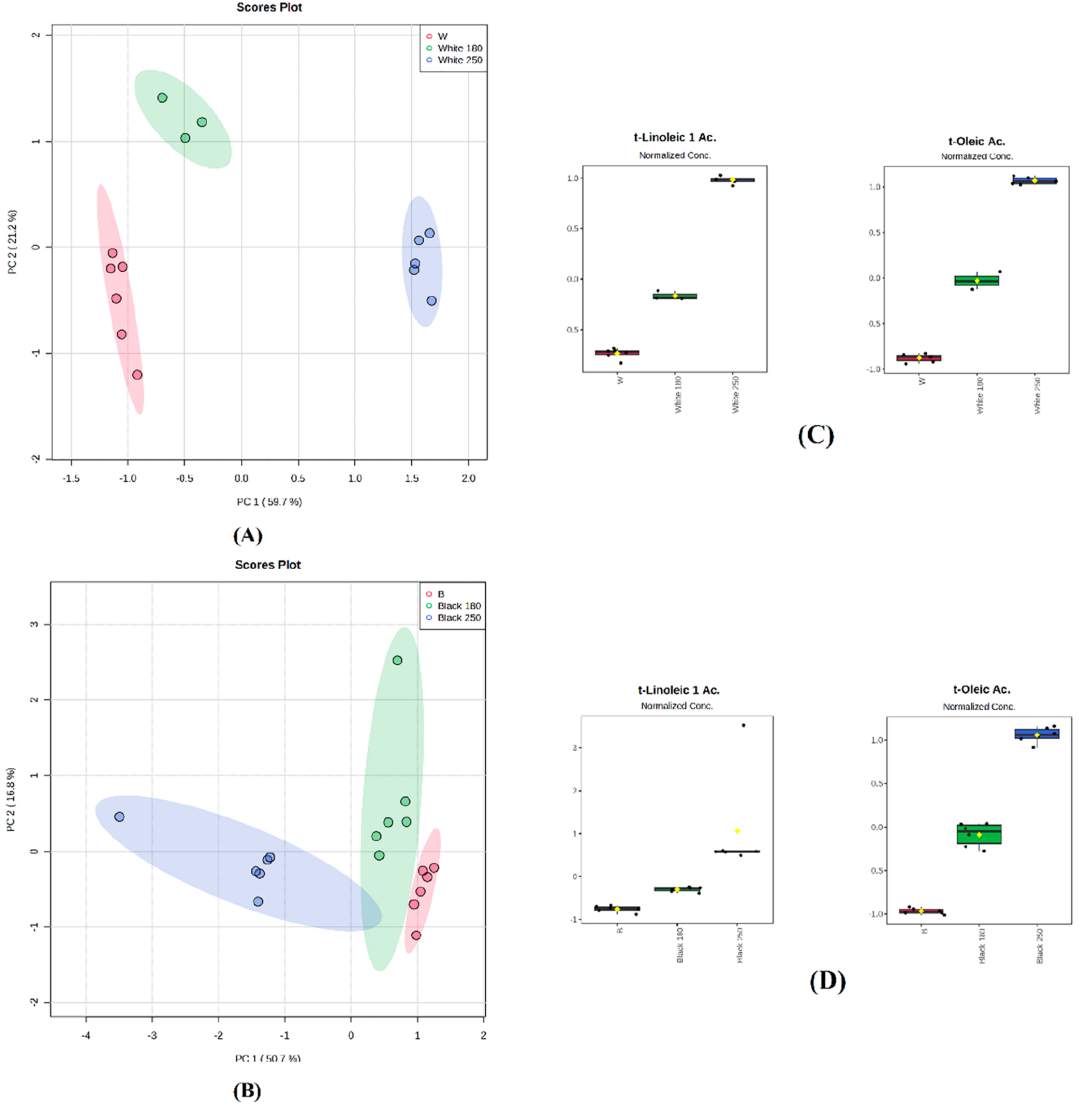
PCA analysis of selected sesame variety subjected to roasting process using fatty acids composition. (A) score plot for white (W) coat color seeds; (B) score plot for black (B) coat color seeds; (C) box plots reporting the variation of *t*-linoleic 1 acid and *t*-oleic acid concentrations in the three different treatments for white coat color seeds: not roasted (red), roasted at 180 °C (green), roasted at 250 °C (blue); (D) box plots reporting the variation of *t*-linoleic 1 acid and *t*-oleic acid concentrations in the three different treatments for black coat color seeds: not roasted (red), roasted at 180 °C (green), roasted at 250 °C (blue).

Moreover, there was an overlap between the heated seeds, while along PC2, an independent cluster of the raw seeds could be seen at the top of the score plot (Figure 4B). Again, the most important variable was sesamol (Figure 4D). These results further support the previous ANOVA analysis, confirming the strong effect of the thermal treatment on the concentration of sesamol. Nevertheless, the PCA highlights a difference between white and black varieties, as the effect of the roasting is less marked for accessions with black seeds. The score plots for the PCA analysis for FAs are shown in Figure 5. Sesame seeds clustered into three groups, each for a thermal treatment, for both white (Figure 5A) and black (Figure 5B). For white seed accessions, PC1 and PC2 accounted for 80.9% of the total variability (Figure 5A). Overall, the *t*-oleic acid and *t*-linoleic 1 acid concentrations increased proportionally according to the temperature applied and were the most important variables for sample clustering (Figure 5C). *t*-Oleic acid is reported to be present in low amounts in untreated varieties, while it is present in far greater concentration when roasting is applied. It reaches the highest concentration when a temperature equal to 250 °C is applied.

For black seeded accessions, PC1 and PC2 accounted for 67.5% of the total variability. *t*-Linoleic 1 acid and *t*-oleic acid were also the variables accounting for clustering (3D). This exploratory analysis highlighted the potential application of chemometrics in the chemical evaluation of sesame components, which is crucial in assessing processing effects to ensure food quality and safety. Chemometrics already has a significant role in food science and food analysis.^37^

From our findings, it clearly emerges that extensive heat treatments lead to unfavorable degradation of nutritional attributes. There was a sesamolin and sesamin reduction, as well as an increase of *trans* fatty acids when elevated temperatures (250 °C) for 25 min were applied. In accordance with this, other studies reported a gradual decline of bioactive components (i.e., sesamolin, phytosterols, total tocopherols) and a great increase of potentially harmful substances, such as polycylic aromatic hydrocarbons, Maillard reaction compounds and *trans* fatty acids, when high temperatures for a prolonged time were applied.^36−39^ In addition, sugar degradation, protein denaturation and lipid oxidation were reported to take place.^39^

Still roasting enhances sensory attributes (flavor, color, texture),^38^ and it leads to the formation of new compounds having antioxidant activity, such as sesamol, undesirable changes may also occur, leading to nutritional losses and formation of potentially toxic compounds. A low level of *trans* UFAs is desirable because their consumption is strongly associated with increased risk of cardio-vascular diseases (CVDs), coronary heart diseases (CHDs) and related mortality, and an increase in the levels of LDL cholesterol.^40,41^ In this regard, WHO strongly recommends that adults and children should reduce trans-fatty acid intake to (or less) 1% of total energy intake, through the replacement of animal fats with oils rich in PUFAs.^33,34^

Therefore, the application of a thermal processing with a milder temperature (up to 180 °C) and a time of less than 20 min would help preserving sesame oil nutritional quality.^38^

In conclusion, in the present study, the correlations among selected quality indicators (i.e., polyphenol content and fatty acid composition) and different color varieties of sesame (white, brown, black) were evaluated, to create an understanding of their interrelations for future breeding improvements. Moreover, the impact on the roasting process on white and black seeded accessions was examined. While further studies confirming our findings and also quantifying the effect of different time/temperature processing conditions are needed, the results of this study pointed out a very diverse polyphenol content and fatty acid unsaturation pattern among the color varieties and after roasting. Therefore, they represent a promising starting point for the field evaluation of the present sesame collection, also using chemometrics analysis to improve interpretation and decision-making, over multiple years.

Whereas it would be desirable to grow varieties with a guaranteed high oil quality, this often proves difficult to achieve, as the polyphenol content and fatty acid composition are greatly influenced by a combination of genetic and environmental factors, worsened by the impact of climate change. From here, the importance to have data also coming from a Mediterranean environment to obtain an evaluation in multilocation trials.

## Supporting Information Available

Complete list of sesame (*Sesamum indicum*) accessions used in agronomical characterization; additional experimental results, including tables reporting sesamolin, sesamin, sesamol, total lignan content, expressed in mg/g, fatty acids composition expressed in % (g/100 g of oil) organized according to sesame seeds colors, and a table reporting the fatty acid composition expressed in % (g/100 g of oil) of selected seeds roasted at 180 and 250 °C (PDF)
