# Supplementary material for: Variability of Fatty Acid Composition and Lignan Content in Sesame Germplasm, and Effect of Roasting
Source: ACS Food Sci Technol. 2023 Oct 2;3(10):1747–58. doi: 10.1021/acsfoodscitech.3c00304 (PMC10594645; doi:10.1021/acsfoodscitech.3c00304)
Supplement: Supplementary file 1 — fs3c00304_si_001.pdf [file fs3c00304_si_001.pdf]

Variability of fatty acid composition and lignan content in sesame germplasm, and effect of roasting.

Eleonora Comini[†], Diego Rubiales[†] and Pierluigi Reveglia[†]

[†] Institute for Sustainable Agriculture, CSIC, Córdoba, 14004, Spain

Supplementay Table 1. List of sesame (*Sesamum indicum*) accessions used in agronomical characterization.

Accession number	Nombre vulgar	Donor gene bank	Country of origin	Improvement status
1	Unknown	IPK	South Korea	Unknown
2	Simsim	IPK	Irak	Unknown
3	Unknown	IPK	Irak	Unknown
4	Unknown	IPK	North Korea	Unknown
5	kunzud	IPK	Tadshikistan	Unknown
6	Unknown	IPK	Tadshikistan	Unknown
7	Unknown	IPK	Tadshikistan	Unknown
8	Unknown	IPK	Irak	Unknown
9	Unknown	IPK	Irak	Unknown
10	Unknown	IPK	Irak	Unknown
11	Unknown	IPK	Bulgaria	Unknown
12	Unknown	IPK	Bulgaria	Unknown
13	JILJILAN	IPK	Yemen	Unknown
14	Unknown	IPK	Yemen	Unknown
15	SISIM	IPK	Yemen	Unknown
16	Unknown	IPK	Yemen	Unknown
17	Unknown	IPK	Yemen	Unknown
18	Unknown	IPK	Yemen	Unknown
19	Unknown	IPK	Yemen	Unknown
20	JILJILAN	IPK	Yemen	Unknown
21	Unknown	IPK	Yemen	Unknown
22	Unknown	IPK	Yemen	Unknown
23	SISUM	IPK	Yemen	Unknown
24	Unknown	IPK	Yemen	Unknown
25	JILJILAN	IPK	Yemen	Unknown
26	Unknown	IPK	Yemen	Unknown
27	Unknown	IPK	Yemen	Unknown

28	Unknown	IPK	Yemen	Unknown
29	SISAM	IPK	Yemen	Unknown
30	Unknown	IPK	Yemen	Unknown
31	SISIM	IPK	Yemen	Unknown
32	SIMSIM	IPK	Yemen	Unknown
33	Unknown	IPK	Yemen	Unknown
34	Unknown	IPK	Yemen	Unknown
35	Unknown	IPK	Yemen	Unknown
36	Unknown	IPK	Yemen	Unknown
37	BLANCO	GRIN-USDA	Texas, USA	Cultivar
38	LLANO	GRIN-USDA	Texas, USA	Cultivar
39	MARGO	GRIN-USDA	Texas, USA	Cultivar
40	ORO	GRIN-USDA	Maryland, USA	Cultivar
41	RIO	GRIN-USDA	Texas, USA	Cultivar
42	Grif 5501	GRIN-USDA	Nepal	Uknown
43	Grif 5502	GRIN-USDA	Nepal	Uknown
44	Grif 5503	GRIN-USDA	Nepal	Uknown
45	Grif 5504	GRIN-USDA	Nepal	Uknown
46	Grif 5505	GRIN-USDA	Nepal	Uknown
47	RENNER NO 1	GRIN-USDA	Texas, USA	Cultivar
48	RENNER COMBINE NO 15	GRIN-USDA	Texas, USA	Cultivar
49	RENNER NO 2	GRIN-USDA	Texas, USA	Cultivar
50	CALINDA	GRIN-USDA	California, USA	Cultivar
51	BACO	GRIN-USDA	Texas, USA	Cultivar
52	PALOMA	GRIN-USDA	Texas, USA	Cultivar
53	T 59022 sel.	GRIN-USDA	Texas, USA	Cultivar
54	T 59326 sel.	GRIN-USDA	Texas, USA	Cultivar
55	T 59108 sel.	GRIN-USDA	Texas, USA	Cultivar
56	T 60073 sel.	GRIN-USDA	Texas, USA	Cultivar
57	T 61325 sel.	GRIN-USDA	Texas, USA	Cultivar

58	T 62046 sel.	GRIN-USDA	Texas, USA	Cultivar
59	T 62281-B-2-B	GRIN-USDA	Texas, USA	Cultivar
60	T 62281-B-6-B	GRIN-USDA	Texas, USA	Cultivar
61	T 61521-B-2-7-B	GRIN-USDA	Texas, USA	Cultivar
62	T 61521-B-2-10-B	GRIN-USDA	Texas, USA	Cultivar
63	T 61226-B-1-1-B	GRIN-USDA	Texas, USA	Cultivar
64	T 61357-B-1-1-B	GRIN-USDA	Texas, USA	Cultivar
65	T 61421-B-1-1-1	GRIN-USDA	Texas, USA	Cultivar
66	T 61424-B-2-7-1	GRIN-USDA	Texas, USA	Cultivar
67	T 61429-B-4-1-2	GRIN-USDA	Texas, USA	Cultivar
68	T 61429-B-4-1-3	GRIN-USDA	Texas, USA	Cultivar
69	T 61429-B-4-2-1	GRIN-USDA	Texas, USA	Cultivar
70	T 61429-B-9-1-1	GRIN-USDA	Texas, USA	Cultivar
71	T 61429-B-9-1-2	GRIN-USDA	Texas, USA	Cultivar
72	T 62276-B-2-1	GRIN-USDA	Texas, USA	Cultivar
73	T 62282-B-2-1	GRIN-USDA	Texas, USA	Cultivar
74	T 62282-B-2-2	GRIN-USDA	Texas, USA	Cultivar
75	T 62282-B-2-3	GRIN-USDA	Texas, USA	Cultivar
76	T 62287-B-1-1	GRIN-USDA	Texas, USA	Cultivar
77	T 62287-B-4-1	GRIN-USDA	Texas, USA	Cultivar
78	T 61521-B-3-3-1	GRIN-USDA	Texas, USA	Cultivar
79	T 61521-B-3-3-4	GRIN-USDA	Texas, USA	Cultivar
80	T 61521-B-3-7-3	GRIN-USDA	Texas, USA	Cultivar
81	T 61521-B-3-8-3	GRIN-USDA	Texas, USA	Cultivar
82	T 61502-25-1-1-1	GRIN-USDA	Texas, USA	Cultivar
83	T 61502-25-1-1-2	GRIN-USDA	Texas, USA	Cultivar
84	T 61502-25-1-3-1	GRIN-USDA	Texas, USA	Cultivar
85	T 61502-25-1-3-2	GRIN-USDA	Texas, USA	Cultivar
86	T 61502-25-1-3-3	GRIN-USDA	Texas, USA	Cultivar
87	T 61502-25-1-5-1	GRIN-USDA	Texas, USA	Cultivar

88	T 61502-25-1-5-4	GRIN-USDA	Texas, USA	Cultivar
89	T 61502-25-1-6-1	GRIN-USDA	Texas, USA	Cultivar
90	T 61502-25-1-6-2	GRIN-USDA	Texas, USA	Cultivar
91	T 61502-25-1-8-3	GRIN-USDA	Texas, USA	Cultivar
92	T 61015-B-64-1-1-1	GRIN-USDA	Texas, USA	Cultivar
93	T 61015-B-64-1-1-2	GRIN-USDA	Texas, USA	Cultivar
94	T 61015-B-64-1-1-3	GRIN-USDA	Texas, USA	Cultivar
95	T 61511-61-1-4-1	GRIN-USDA	Texas, USA	Cultivar
96	T 61502-25-2	GRIN-USDA	Texas, USA	Cultivar
97	T 61502-25-3	GRIN-USDA	Texas, USA	Cultivar
98	T 58546-B-1-1-1-1-1	GRIN-USDA	Texas, USA	Cultivar
99	T 58564-B-B-1-3-1-3-1	GRIN-USDA	Texas, USA	Cultivar
100	T 58564-B-B-1-1-1-2-1	GRIN-USDA	Texas, USA	Cultivar
101	T 591000-B-1-1-3-3-4-1	GRIN-USDA	Texas, USA	Cultivar
102	T 59260-B-1-1-4-2-1	GRIN-USDA	Texas, USA	Cultivar
103	T 61066-B-1-1-1	GRIN-USDA	Texas, USA	Cultivar
104	T 61178-B-4-5-1	GRIN-USDA	Texas, USA	Cultivar
105	T 61499-B-1-2-1	GRIN-USDA	Texas, USA	Cultivar
106	T 61357-B-1-2-1	GRIN-USDA	Texas, USA	Cultivar
107	T 62130-B-1-1	GRIN-USDA	Texas, USA	Cultivar
108	T 62335-B1-1-B-1	GRIN-USDA	Texas, USA	Cultivar
109	T 62396-2-3-1	GRIN-USDA	Texas, USA	Cultivar
110	T 63039-B-3-1	GRIN-USDA	Texas, USA	Cultivar
111	Unknown	GRIN-USDA	Venezuela	Unknown
112	Criollo	GRIN-USDA	Venezuela	Unknown
113	Guatemala	GRIN-USDA	Venezuela	Unknown
114	Hawaii-Blanco	GRIN-USDA	Venezuela	Unknown
115	Hawaii	GRIN-USDA	Venezuela	Unknown
116	Mejico	GRIN-USDA	Venezuela	Unknown
117	Nicaragua (Hunt)	GRIN-USDA	Venezuela	Unknown

118	Nicaragua (Horvilleur)	GRIN-USDA	Venezuela	Unknown
119	Venezuela 51	GRIN-USDA	Venezuela	Unknown
120	1-2-5-1-3	GRIN-USDA	Mexico	Unknown
121	3-4-6-6-1	GRIN-USDA	Mexico	Unknown
122	5-1-4-3-5	GRIN-USDA	Mexico	Unknown
123	7-3-5-4-2	GRIN-USDA	Mexico	Unknown
124	8-2-6-3-1	GRIN-USDA	Mexico	Unknown
125	Unknown	GRIN-USDA	Mexico	Unknown
126	10-3-6-3-5	GRIN-USDA	Mexico	Unknown
127	Unknown	GRIN-USDA	Mexico	Unknown
128	14-4-5-2-2	GRIN-USDA	Mexico	Unknown
129	25-2-5-6-6	GRIN-USDA	Mexico	Unknown
130	34-3-5-1-4	GRIN-USDA	Mexico	Unknown
131	Unknown	GRIN-USDA	China	Unknown
132	Unknown	GRIN-USDA	India	Unknown
133	S.1.53	GRIN-USDA	India	Unknown
134	Unknown	GRIN-USDA	India	Unknown
135	Unknown	GRIN-USDA	India	Unknown
136	IP 7	GRIN-USDA	India	Unknown
137	Unknown	GRIN-USDA	India	Unknown
138	IP 19	GRIN-USDA	India	Unknown
139	Unknown	GRIN-USDA	India	Unknown
140	IP 24	GRIN-USDA	India	Unknown
141	IP 29	GRIN-USDA	India	Unknown
142	IP 30	GRIN-USDA	India	Unknown
143	IP Hybrid 1205-18	GRIN-USDA	India	Unknown
144	Unknown	GRIN-USDA	China	Unknown
145	Unknown	GRIN-USDA	China	Unknown
146	Unknown	GRIN-USDA	China	Unknown
147	Unknown	GRIN-USDA	China	Unknown

148	Unknown	GRIN-USDA	China	Unknown
149	Unknown	GRIN-USDA	China	Unknown
150	Unknown	GRIN-USDA	China	Unknown
151	Unknown	GRIN-USDA	China	Unknown
152	Unknown	GRIN-USDA	China	Unknown
153	Unknown	GRIN-USDA	China	Unknown
154	Unknown	GRIN-USDA	China	Unknown
155	Unknown	GRIN-USDA	China	Unknown
156	Unknown	GRIN-USDA	China	Unknown
157	Unknown	GRIN-USDA	China	Unknown
158	Unknown	GRIN-USDA	China	Unknown
159	Unknown	GRIN-USDA	China	Unknown
160	Unknown	GRIN-USDA	China	Unknown
161	Unknown	GRIN-USDA	China	Unknown
162	Unknown	GRIN-USDA	China	Unknown
163	Unknown	GRIN-USDA	China	Unknown
164	Unknown	GRIN-USDA	China	Unknown
165	Unknown	GRIN-USDA	China	Unknown
166	Unknown	GRIN-USDA	China	Unknown
167	Unknown	GRIN-USDA	China	Unknown
168	Unknown	GRIN-USDA	China	Unknown
169	Unknown	GRIN-USDA	China	Unknown
170	Unknown	GRIN-USDA	China	Unknown
171	Venezuela-51	GRIN-USDA	Venezuela	Unknown
172	Venezuela-52	GRIN-USDA	Venezuela	Unknown
173	Crillo	GRIN-USDA	Venezuela	Unknown
174	44-575	GRIN-USDA	Venezuela	Unknown
175	44-473	GRIN-USDA	Venezuela	Unknown
176	43-80	GRIN-USDA	Venezuela	Unknown
177	Unknown	GRIN-USDA	Venezuela	Unknown

178	Unknown	GRIN-USDA	China	Unknown
179	Unknown	GRIN-USDA	China	Unknown
180	Unknown	GRIN-USDA	China	Unknown
181	Unknown	GRIN-USDA	China	Unknown
182	Unknown	GRIN-USDA	China	Unknown
183	Unknown	GRIN-USDA	China	Unknown
184	Unknown	GRIN-USDA	China	Unknown
185	Unknown	GRIN-USDA	China	Unknown
186	Unknown	GRIN-USDA	China	Unknown
187	Unknown	GRIN-USDA	China	Unknown
188	Unknown	GRIN-USDA	China	Unknown
189	Unknown	GRIN-USDA	China	Unknown
190	Unknown	GRIN-USDA	China	Unknown
191	Unknown	GRIN-USDA	China	Unknown
192	Unknown	GRIN-USDA	China	Unknown
193	Unknown	GRIN-USDA	China	Unknown
194	Unknown	GRIN-USDA	China	Unknown
195	Unknown	GRIN-USDA	China	Unknown
196	Unknown	GRIN-USDA	China	Unknown
197	Kyorgii Do	GRIN-USDA	Korea	Unknown
198	Unknown	GRIN-USDA	China	Unknown
199	1041	GRIN-USDA	Guatemala	Unknown
200	Til	GRIN-USDA	India	Unknown
201	Yellu	GRIN-USDA	India	Unknown
202	Til	GRIN-USDA	India	Unknown
203	Til	GRIN-USDA	India	Unknown
204	Til	GRIN-USDA	India	Unknown
205	9071	GRIN-USDA	India	Unknown
206	Til	GRIN-USDA	India	Unknown
207	Til	GRIN-USDA	India	Unknown

208	Unknown	GRIN-USDA	Maryland, USA	Unknown
209	No.26	GRIN-USDA	Guatemala	Unknown
210	Susam	GRIN-USDA	Turkey	Unknown
211	Til	GRIN-USDA	India	Unknown
212	Til	GRIN-USDA	India	Unknown
213	Til	GRIN-USDA	India	Unknown
214	Unknown	GRIN-USDA	Turkey	Unknown
215	418	GRIN-USDA	Turkey	Unknown
216	Susam	GRIN-USDA	Turkey	Unknown
217	Susam	GRIN-USDA	Turkey	Unknown
218	1496	GRIN-USDA	Turkey	Unknown
219	1523	GRIN-USDA	Turkey	Unknown
220	1532	GRIN-USDA	Turkey	Unknown
221	1644	GRIN-USDA	Turkey	Unknown
222	1665	GRIN-USDA	Turkey	Unknown
223	1671	GRIN-USDA	Turkey	Unknown
224	1678	GRIN-USDA	Turkey	Unknown
225	1687	GRIN-USDA	Turkey	Unknown
226	1903	GRIN-USDA	Turkey	Unknown
227	1904	GRIN-USDA	Turkey	Unknown
228	1918	GRIN-USDA	Turkey	Unknown
229	Kivrak Susam	GRIN-USDA	Turkey	Unknown
230	Gec Yetisen	GRIN-USDA	Turkey	Unknown
231	2000	GRIN-USDA	Turkey	Unknown
232	2018	GRIN-USDA	Turkey	Unknown
233	2026	GRIN-USDA	Turkey	Unknown
234	2033	GRIN-USDA	Turkey	Unknown
235	2145	GRIN-USDA	Turkey	Unknown
236	2226	GRIN-USDA	Turkey	Unknown
237	2307	GRIN-USDA	Turkey	Unknown

238	2514	GRIN-USDA	Turkey	Unknown
239	2540	GRIN-USDA	Turkey	Unknown
240	2541	GRIN-USDA	Turkey	Unknown
241	Unknown	GRIN-USDA	Turkey	Unknown
242	2609	GRIN-USDA	Turkey	Unknown
243	2665	GRIN-USDA	Turkey	Unknown
244	2703	GRIN-USDA	Turkey	Unknown
245	2847	GRIN-USDA	Turkey	Unknown
246	2838	GRIN-USDA	Turkey	Unknown
247	Unknown	GRIN-USDA	Turkey	Unknown
248	2875	GRIN-USDA	Turkey	Unknown
249	3005	GRIN-USDA	Turkey	Unknown
250	3030	GRIN-USDA	Turkey	Unknown
251	Unknown	GRIN-USDA	Maryland, USA	Unknown
252	3221	GRIN-USDA	Turkey	Unknown
253	3268	GRIN-USDA	Turkey	Unknown
254	3286	GRIN-USDA	Turkey	Unknown
255	3310	GRIN-USDA	Turkey	Unknown
256	3435	GRIN-USDA	Turkey	Unknown
257	3439	GRIN-USDA	Turkey	Unknown
258	3497	GRIN-USDA	Turkey	Unknown
259	3505	GRIN-USDA	Turkey	Unknown
260	3769	GRIN-USDA	Turkey	Unknown
261	3830	GRIN-USDA	Turkey	Unknown
262	Unknown	GRIN-USDA	Turkey	Unknown
263	3832	GRIN-USDA	Turkey	Unknown
264	7527	GRIN-USDA	Turkey	Unknown
265	8669	GRIN-USDA	Turkey	Unknown
266	9734-A	GRIN-USDA	India	Unknown
267	9739	GRIN-USDA	India	Unknown

268	9752	GRIN-USDA	India	Unknown
269	9769	GRIN-USDA	India	Unknown
270	9780	GRIN-USDA	India	Unknown
271	9797	GRIN-USDA	India	Unknown
272	9817	GRIN-USDA	India	Unknown
273	9823	GRIN-USDA	India	Unknown
274	9825	GRIN-USDA	India	Unknown
275	9830	GRIN-USDA	India	Unknown
276	9835	GRIN-USDA	India	Unknown
277	9842	GRIN-USDA	India	Unknown
278	9843	GRIN-USDA	India	Unknown
279	9846	GRIN-USDA	India	Unknown
280	9851	GRIN-USDA	India	Unknown
281	7761	GRIN-USDA	Turkey	Unknown
282	8422	GRIN-USDA	Turkey	Unknown
283	8751	GRIN-USDA	Turkey	Unknown
284	Til	GRIN-USDA	India	Unknown
285	Til	GRIN-USDA	India	Unknown
286	Til	GRIN-USDA	India	Unknown
287	Til	GRIN-USDA	India	Unknown
288	Til	GRIN-USDA	India	Unknown
289	Til	GRIN-USDA	Nepal	Unknown
290	Til	GRIN-USDA	India	Unknown
291	Til	GRIN-USDA	India	Unknown
292	Til	GRIN-USDA	India	Unknown
293	Til	GRIN-USDA	India	Unknown
294	5452	GRIN-USDA	Turkey	Unknown
295	5984	GRIN-USDA	Turkey	Unknown
296	6079	GRIN-USDA	Turkey	Unknown
297	5545	GRIN-USDA	Turkey	Unknown

298	5605	GRIN-USDA	Turkey	Unknown
299	9550	GRIN-USDA	Turkey	Unknown
300	5196	GRIN-USDA	Turkey	Unknown
301	6315	GRIN-USDA	Turkey	Unknown
302	10160	GRIN-USDA	Turkey	Unknown
303	10185	GRIN-USDA	Turkey	Unknown
304	10187	GRIN-USDA	Turkey	Unknown
305	10200	GRIN-USDA	Turkey	Unknown
306	10201	GRIN-USDA	Turkey	Unknown
307	4521	GRIN-USDA	Turkey	Unknown
308	5817	GRIN-USDA	Turkey	Unknown
309	5817	GRIN-USDA	Turkey	Unknown
310	5934	GRIN-USDA	Turkey	Unknown
311	9851	GRIN-USDA	Iraq	Unknown
312	10085	GRIN-USDA	Turkey	Unknown
313	10098	GRIN-USDA	Turkey	Unknown
314	10222	GRIN-USDA	Turkey	Unknown
315	10361	GRIN-USDA	Turkey	Unknown
316	Til	GRIN-USDA	India	Unknown
317	Til	GRIN-USDA	India	Unknown
318	Til	GRIN-USDA	India	Unknown
319	Til	GRIN-USDA	India	Unknown
320	Safad	GRIN-USDA	India	Unknown
321	Til	GRIN-USDA	India	Unknown
322	Unknown	GRIN-USDA	India	Unknown
323	Tal	GRIN-USDA	India	Unknown
324	Til	GRIN-USDA	India	Unknown
325	Unknown	GRIN-USDA	India	Unknown
326	Til	GRIN-USDA	India	Unknown
327	Kala Til	GRIN-USDA	India	Unknown

328	Tal	GRIN-USDA	India	Unknown
329	Tal	GRIN-USDA	India	Unknown
330	Tal	GRIN-USDA	India	Unknown
331	Tal	GRIN-USDA	India	Unknown
332	Tal	GRIN-USDA	India	Unknown
333	Til	GRIN-USDA	India	Unknown
334	Til	GRIN-USDA	India	Unknown
335	Tal	GRIN-USDA	India	Unknown
336	Tal	GRIN-USDA	India	Unknown
337	Til	GRIN-USDA	India	Unknown
338	Til	GRIN-USDA	India	Unknown
339	Til	GRIN-USDA	India	Unknown
340	Til	GRIN-USDA	India	Unknown
341	Tal	GRIN-USDA	India	Unknown
342	Tal	GRIN-USDA	India	Unknown
343	8620	GRIN-USDA	India	Unknown
344	8621	GRIN-USDA	India	Unknown
345	8622	GRIN-USDA	India	Unknown
346	8623	GRIN-USDA	India	Unknown
347	8624	GRIN-USDA	India	Unknown
348	8625	GRIN-USDA	India	Unknown
349	10470	GRIN-USDA	Turkey	Unknown
350	10559	GRIN-USDA	Turkey	Unknown
351	10552	GRIN-USDA	Turkey	Unknown
352	Tal	GRIN-USDA	India	Unknown
353	Tal	GRIN-USDA	India	Unknown
354	11428	GRIN-USDA	India	Unknown
355	Tal	GRIN-USDA	India	Unknown
356	11164	GRIN-USDA	India	Unknown
357	11426	GRIN-USDA	India	Unknown

358	11427	GRIN-USDA	India	Unknown
359	Tal	GRIN-USDA	India	Unknown
360	Tal	GRIN-USDA	India	Unknown
361	Tal	GRIN-USDA	India	Unknown
362	Tal	GRIN-USDA	India	Unknown
363	Unknown	GRIN-USDA	India	Unknown
364	Tili	GRIN-USDA	India	Unknown
365	Tal	GRIN-USDA	India	Unknown
366	11183	GRIN-USDA	India	Unknown
367	11281	GRIN-USDA	India	Unknown
368	11430	GRIN-USDA	India	Unknown
369	Tili	GRIN-USDA	India	Unknown
370	Tal	GRIN-USDA	India	Unknown
371	11310	GRIN-USDA	India	Unknown
372	Tal	GRIN-USDA	India	Unknown
373	Til	GRIN-USDA	India	Unknown
374	Til	GRIN-USDA	India	Unknown
375	Dumboy	GRIN-USDA	Liberia	Unknown
376	Venezuela 51	GRIN-USDA	Venezuela	Unknown
377	Venezuela 52	GRIN-USDA	Venezuela	Unknown
378	43-80	GRIN-USDA	Venezuela	Unknown
379	Unknown	GRIN-USDA	Nigeria	Unknown
380	Unknown	GRIN-USDA	Nigeria	Unknown
381	Unknown	GRIN-USDA	Nigeria	Unknown
382	Lingnga	GRIN-USDA	Philippines	Unknown
383	No.1	GRIN-USDA	Cameroon	Unknown
384	No.2	GRIN-USDA	Cameroon	Unknown
385	Unknown	GRIN-USDA	Congo	Unknown
386	Unknown	GRIN-USDA	China	Unknown
387	Long Capsule	GRIN-USDA	China	Unknown

388	Par-wang-pien	GRIN-USDA	China	Unknown
389	SESACO 2A	GRIN-USDA	USA	Cultivar
390	L-7	GRIN-USDA	India	Unknown
391	L-16	GRIN-USDA	India	Unknown
392	Boke-Taung	GRIN-USDA	Myanmar	Unknown
393	Byat-Ka-Lay	GRIN-USDA	Myanmar	Unknown
394	Unknown	GRIN-USDA	Myanmar	Unknown
395	Pa-Day-Tha	GRIN-USDA	Myanmar	Unknown
396	Poke-Pyi	GRIN-USDA	Myanmar	Unknown
397	Ye-Gyaw	GRIN-USDA	Myanmar	Unknown
398	Unknown	GRIN-USDA	Jordan	Unknown
399	Unknown	GRIN-USDA	Turkey	Unknown
400	Unknown	GRIN-USDA	Turkey	Unknown
401	Unknown	GRIN-USDA	Turkey	Unknown
402	Unknown	GRIN-USDA	Turkey	Unknown
403	Unknown	GRIN-USDA	India	Unknown
404	Unknown	GRIN-USDA	Japan	Unknown
405	Unknown	GRIN-USDA	Japan	Unknown
406	Unknown	GRIN-USDA	Japan	Unknown
407	Unknown	GRIN-USDA	Japan	Unknown
408	Unknown	GRIN-USDA	Japan	Unknown
409	Unknown	GRIN-USDA	Ethiopia	Unknown
410	Unknown	GRIN-USDA	Somalia	Unknown
411	Unknown	GRIN-USDA	Afghanistan	Unknown
412	Unknown	GRIN-USDA	Afghanistan	Unknown
413	Unknown	GRIN-USDA	Afghanistan	Unknown
414	Unknown	GRIN-USDA	Afghanistan	Unknown
415	Unknown	GRIN-USDA	Afghanistan	Unknown
416	13086	GRIN-USDA	Afghanistan	Unknown
417	13130	GRIN-USDA	Afghanistan	Unknown

418	13365	GRIN-USDA	India	Unknown
419	13530	GRIN-USDA	India	Unknown
420	Local No.8	GRIN-USDA	Iraq	Unknown
421	Mosol No.28	GRIN-USDA	Iraq	Unknown
422	Rousiun	GRIN-USDA	Iraq	Unknown
423	White Jiza	GRIN-USDA	Iraq	Unknown
424	13743	GRIN-USDA	India	Unknown
425	13744	GRIN-USDA	India	Unknown
426	13934	GRIN-USDA	Pakistan	Unknown
427	Konjet	GRIN-USDA	Afghanistan	Unknown
428	Unknown	GRIN-USDA	Afghanistan	Unknown
429	Kunjet	GRIN-USDA	Afghanistan	Unknown
430	Konjet	GRIN-USDA	Afghanistan	Unknown
431	Konjet	GRIN-USDA	Iran	Unknown
432	Konjet	GRIN-USDA	Iran	Unknown
433	Konjet	GRIN-USDA	Iran	Unknown
434	Konjet	GRIN-USDA	Iran	Unknown
435	Konjet	GRIN-USDA	Iran	Unknown
436	Konjet	GRIN-USDA	Iran	Unknown
437	Konjed	GRIN-USDA	Afghanistan	Unknown
438	Til-Monsoon	GRIN-USDA	India	Unknown
439	Konjet	GRIN-USDA	Afghanistan	Unknown
440	1274	GRIN-USDA	Afghanistan	Unknown
441	Konjet	GRIN-USDA	Afghanistan	Unknown
442	No.45	GRIN-USDA	Lybia	Unknown
443	No.64A	GRIN-USDA	Lybia	Unknown
444	Unknown	GRIN-USDA	Lybia	Unknown
445	Inamar	GRIN-USDA	Venezuela	Unknown
446	Morada	GRIN-USDA	Venezuela	Unknown
447	382	GRIN-USDA	Ethiopia	Unknown

448	383	GRIN-USDA	Ethiopia	Unknown
449	Konjet	GRIN-USDA	Iran	Unknown
450	950-DCE-Sm.-S.I.0500	GRIN-USDA	Argentina	Unknown
451	950-DCE-Sm.-V52-03	GRIN-USDA	Argentina	Unknown
452	Conjet	GRIN-USDA	Iran	Unknown
453	Almadnagor White	GRIN-USDA	Mozambique	Unknown
454	Branco	GRIN-USDA	Mozambique	Unknown
455	Satora Brown	GRIN-USDA	Mozambique	Unknown
456	Viragaon Brown	GRIN-USDA	Mozambique	Unknown
457	Viragaon Brown	GRIN-USDA	Mozambique	Unknown
458	Viragaon Brown	GRIN-USDA	Mozambique	Unknown
459	Tainan Black No.1	GRIN-USDA	Taiwan	Unknown
460	Tainan Black No.2	GRIN-USDA	Taiwan	Unknown
461	Tainan Black Selected	GRIN-USDA	Taiwan	Unknown
462	Tainan White No.1	GRIN-USDA	Taiwan	Unknown
463	Tainan White No.2	GRIN-USDA	Taiwan	Unknown
464	Unknown	GRIN-USDA	Japan	Unknown
465	Unknown	GRIN-USDA	Nigeria	Unknown
466	K932	GRIN-USDA	Iraq	Unknown
467	N.P.6	GRIN-USDA	Iraq	Unknown
468	No.1	GRIN-USDA	South America	Unknown
469	PRECOZ	GRIN-USDA	Nicaragua	Unknown
470	No.3	GRIN-USDA	Texas, USA	Unknown
471	INSTITUTO NO.1	GRIN-USDA	Mexico	Unknown
472	INSTITUTO NO.3	GRIN-USDA	Mexico	Unknown
473	INSTITUTO NO.8	GRIN-USDA	Mexico	Unknown
474	INSTITUTO NO. 10-3	GRIN-USDA	Mexico	Unknown
475	INSTITUTO NO. 15	GRIN-USDA	Mexico	Unknown
476	INSTITUTO NO. 108	GRIN-USDA	Mexico	Unknown
477	92	GRIN-USDA	Texas, USA	Unknown

478	94	GRIN-USDA	Texas, USA	Unknown
479	98	GRIN-USDA	Texas, USA	Unknown
480	101	GRIN-USDA	Texas, USA	Unknown
481	103	GRIN-USDA	Texas, USA	Unknown
482	105	GRIN-USDA	Texas, USA	Unknown
483	108	GRIN-USDA	Texas, USA	Unknown
484	113	GRIN-USDA	Texas, USA	Unknown
485	121	GRIN-USDA	Texas, USA	Unknown
486	124	GRIN-USDA	Texas, USA	Unknown
487	126	GRIN-USDA	Texas, USA	Unknown
488	128	GRIN-USDA	Texas, USA	Unknown
489	132	GRIN-USDA	Texas, USA	Unknown
490	133	GRIN-USDA	Texas, USA	Unknown
491	135	GRIN-USDA	Texas, USA	Unknown
492	150	GRIN-USDA	Texas, USA	Unknown
493	Eilabun	GRIN-USDA	Israel	Unknown
494	4/b	GRIN-USDA	Israel	Unknown
495	Unknown	GRIN-USDA	Israel	Unknown
496	Unknown	GRIN-USDA	Israel	Unknown
497	Unknown	GRIN-USDA	Israel	Unknown
498	355	GRIN-USDA	India	Unknown
499	Unknown	GRIN-USDA	India	Unknown
500	504	GRIN-USDA	Nepal	Unknown
501	515	GRIN-USDA	Nepal	Unknown
502	537	GRIN-USDA	Nepal	Unknown
503	548	GRIN-USDA	Nepal	Unknown
504	556	GRIN-USDA	India	Unknown
505	564	GRIN-USDA	Nepal	Unknown
506	579	GRIN-USDA	Nepal	Unknown
507	Sesamo blanco	Purchased in a market	Thailand	Unknown

508	Sesamo negro	Purchased in a market	Thailand	Unknown
509	Shirogoma	Purchased in a market	Unknown	Unknown
510	Kurogoma	Purchased in a market	Unknown	Unknown

Supplementary Table 2. Sesamolin, sesamin, sesamol and total lignan content, expressed in mg/g, in white sesame seeds.

Accession	Sesamolin	Sesamin	Sesamol	Total
54	3.27±0.23*	2.59±0.51	0.02±0.01	5.88
56	1.21±0.17	2.57±0.17	0.01±0.01	3.79
67	5.20±0.78	2.10±0.39	0.03±0.01	7.33
76	3.90 ± 0.43	6.40±0.79	0.02±0.01	10.32
79	2.60 ±0.21	2.55±0.55	0.02±0.00	5.17
114	2.50±0.85	3.01±0.74	0.07±0.02	5.58
118	3.15±0.70	6.27±1.33	0.08±0.01	9.50
123	2.13±0.44	2.10±0.30	0.06±0.03	4.29
173	4.50±0.32	2.15±0.48	0.05±0.01	6.70
224	3.13±0.09	6.87±1.43	0.05±0.01	10.05
246	0.69±0.11	1.09±0.40	0.03±0.01	1.81
285	3.82±0.39	4.93±0.05	0.07±0.01	8.82
292	2.47±0.25	5.29±0.84	0.06±0.01	7.82
293	1.22± 0.24	2.43±1.31	0.03±0.02	3.68
315	0.97±0.25	0.37±0.18	0.08±0.02	1.42
317	1.89±0.17	1.24±0.07	0.07±0.00	3.20
318	2.92±0.45	7.03±0.07	0.02±0.00	9.97
324	0.76±0.05	2.27±0.86	0.03±0.01	3.06
332	5.28±0.91	6.27±0.42	0.03±0.00	11.58
393	2.82±0.14	1.66±0.77	0.12±0.06	4.60
473	3.86±0.48	1.86±0.59	0.13±0.01	5.85

*Mean ± SD

Supplementary Table 3. Sesamolin, sesamin, sesamol and total lignan content, expressed in mg/g, in brown sesame seeds.

Sample	Sesamolin	Sesamin	Sesamol	Total
87	2.38±0.47*	2.20±0.67	0.03±0.01	4.61
93	2.10±0.28	1.47±0.31	0.01±0.01	3.58
110	2.01±0.15	2.78±0.17	0.08±0.02	4.87
167	7.04±0.61	7.05±1.83	0.09±0.01	14.18
168	3.61±0.04	3.71±0.82	0.07±0.02	7.39
176	2.35±0.45	2.76±0.27	0.17±0.01	5.28
218	1.76±0.22	5.97±1.29	0.02±0.01	7.75
228	1.45±0.37	5.51±1.30	0.03±0.01	6.99
258	0.60±0.07	3.76±0.56	0.01±0.00	4.37
265	1.22±0.01	5.70±0.50	0.02±0.02	6.94
314	0.64±0.13	5.74±0.56	0.03±0.01	6.41
427	2.81±0.35	5.89±0.16	0.03±0.00	8.73
483	4.97±0.51	7.54±0.62	0.13±0.03	12.64
484	6.07±0.60	6.21±2.82	0.10±0.02	12.38

*Mean ± SD

Supplementary Table 4. Sesamolin, sesamin, sesamol and total lignan content, expressed in mg/g, in black sesame seeds.

Sample	Sesamolin	Sesamin	Sesamol	Total
141	2.55±0.12	3.80±0.15	0.09±0.00	6.44
152	3.76±0.17	2.26±0.05	0.17±0.01	6.19
153	4.83±0.15	5.27±0.17	0.04±0.01	10.14
157	3.69±0.52	4.07±0.26	0.11±0.01	7.87
161	6.28±0.19	4.56±0.34	0.06±0.01	10.9
170	1.17±0.17	1.06±0.08	0.04±0.02	2.27
216	1.67±0.37	4.34±0.10	0.11±0.00	6.12
271	2.36±0.34	2.72±0.61	0.08±0.03	5.16
282	0.72±0.13	2.45±1.73	0.08±0.03	3.25
284	3.19±0.08	4.84±0.13	0.04±0.01	8.07
302	2.59±0.22	8.03±0.58	0.13±0.01	10.75
329	2.80±0.03	5.31±3.40	0.17±0.01	8.28
358	1.74±0.17	3.36±0.58	0.12±0.01	5.22
403	2.16±0.31	8.14±0.10	0.11±0.00	10.41
463	5.14±0.65	4.88±0.21	0.11±0.02	10.13
508	6.35±0.08	8.72±0.55	0.18±0.01	15.25

*Mean ± SD.

Suplementary Table 5. Fatty acid composition expressed in % (g/100 g of oil) of white sesame seeds.

Variety	Myristic acid	Palmitic acid	Palmitoleic 1 acid (ω7)	Palmitoleic 2 acid (ω7)	Margaric acid	Margaroleic acid	Stearic acid	*t-Oleic acid	Oleic acid (ω-7+ ω-9)	t-Linoleic 1 acid	Linoleic acid	Linolenic acid	Arachidic acid	Gadoleic acid	Behenic acid	Lignoceric acid
54	0.02±0.001	9.70±0.47	0.05±0.01	0.15±0.01	0.12±0.01	0.06±0.00	5.51±0.22	0.02±0.002	47.36±1.50^{abc}	0.04±0.003	35.62±1.30	0.35±0.03	0.60±0.02	0.18±0.01	0.13±0.01	0.09±0.004
56	0.02±0.002	8.43±0.19	0.04±0.002	0.10±0.01	0.12±0.01	0.06±0.001	5.65±0.60	0.02±0.002	45.20±2.48^{abc}	0.04±0.001	38.91±3.20	0.37±0.05	0.61±0.04	0.21±0.00	0.13±0.00	0.09±0.003
67	0.02±0.003	9.25±0.41	0.05±0.01	0.13±0.001	0.11±0.00	0.06±0.002	6.16±0.29	0.02±0.002	51.42±0.81^{ab}	0.03±0.002	31.37±0.85	0.30±0.01	0.65±0.02	0.20±0.01	0.13±0.00	0.09± 0.003
76	0.03±0.001	9.25±0.70	0.04±0.01	0.11±0.02	0.11±0.01	0.06±0.001	6.27±0.84	0.02±0.008	47.89±7.01^{abc}	0.04±0.01	34.74±7.28	0.32±0.03	0.69±0.05	0.19±0.02	0.15±0.00	0.10±0.01
79	0.02±0.001	8.13±0.19	0.04±0.01	0.08±0.002	0.12±0.00	0.06±0.01	5.79±0.66	0.02±0.006	50.60±1.69^{b}	0.03±0.002	33.71±2.51	0.32±0.02	0.64±0.05	0.21±0.00	0.14±0.00	0.10±0.01
114	0.02±0.001	8.45±0.16	0.05±0.01	0.10±0.01	0.10±0.02	0.05±0.01	6.13±0.81	0.02±0.004	45.62±4.04^{abc}	0.04±0.002	37.98±4.83	0.40±0.06	0.63±0.07	0.20±0.00	0.12±0.01	0.08±0.01
118	0.02±0.002	9.25±0.14	0.03±0.01	0.11±0.01	0.10±0.01	0.05±0.002	5.76±0.46	0.02±0.005	45.94±4.21^{abc}	0.04±0.01	37.25±4.60	0.30±0.01	0.66±0.05	0.20±0.01	0.15±0.01	0.10±0.01
123	0.02±0.002	8.61±0.44	0.05±0.01	0.10±0.01	0.12±0.00	0.06±0.002	5.99±0.35	0.02±0.004	49.80±4.62^{abc}	0.04±0.002	33.83±4.54	0.29±0.03	0.66±0.04	0.20±0.01	0.13±0.00	0.09±0.01
173	0.02±0.001	8.49±0.10	0.03±0.001	0.10±0.003	0.10±0.01	0.05±0.003	6.54±0.30	0.02±0.003	48.80±0.78^{abc}	0.04±0.003	34.51±1.19	0.30±0.0.1	0.62±0.02	0.19±0.00	0.11±0.00	0.08±0.003
224	0.02±0.002	9.59±0.41	0.03±0.01	0.12±0.01	0.08±0.01	0.04±0.001	5.94±0.38	0.02±0.002	44.34±1.86^{abc}	0.04±0.004	38.45±1.89	0.27±0.01	0.65±0.04	0.18±0.01	0.14±0.01	0.10±0.001
246	0.02±0.003	8.78±0.23	0.06±0.01	0.11±0.01	0.12±0.01	0.06±0.01	5.84±0.22	0.02±0.001	48.07±2.27^{abc}	0.04±0.003	35.32±2.38	0.48±0.09	0.66±0.02	0.21±0.01	0.14±0.00	0.09±0.002
285	0.02±0.001	9.25±0.33	0.03±0.001	0.12±0.01	0.08±0.01	0.04±0.01	5.38±0.89	0.02±0.001	45.17±3.04^{abc}	0.04±0.001	38.54±3.64	0.28±0.03	0.62±0.07	0.20±0.01	0.14±0.00	0.10±0.001
292	0.02±0.002	9.74±0.31	0.04±0.002	0.12±0.01	0.10±0.01	0.05±0.01	5.80±0.43	0.02±0.023	44.03±2.29^{abc}	0.04±0.002	38.74±2.64	0.26±0.01	0.63±0.04	0.18±0.00	0.13±0.01	0.10±0.001
293	0.02±0.001	10.47±0.25	0.03±0.001	0.13±0.01	0.06±0.01	0.03±0.001	4.80±0.42	0.02±0.015	43.40±0.98^{bc}	0.04±0.003	39.79±0.86	0.25±0.03	0.56±0.02	0.18±0.00	0.13±0.01	0.09±0.001
315	0.02±0.001	9.75±0.27	0.04±0.002	0.12±0.001	0.12±0.01	0.06±0.001	5.66±0.19	0.02±0.004	45.56±1.82^{abc}	0.04±0.004	37.26±2.23	0.29±0.02	0.64±0.03	0.19±0.01	0.13±0.01	0.10±0.001
317	0.02±0.001	10.30±0.12	0.04±0.01	0.14±0.001	0.09±0.01	0.05±0.01	5.51±0.37	0.02±0.003	44.04±0.68^{abc}	0.04±0.002	38.43±1.01	0.31±0.04	0.61±0.03	0.18±0.00	0.13±0.00	0.09±0.003
318	0.02±0.002	9.89±0.26	0.03±0.001	0.13±0.01	0.09±0.01	0.05±0.002	5.32±0.34	0.02±0.001	45.47±0.66^{abc}	0.04±0.004	37.79±1.20	023±0.01	0.56±0.03	0.17±0.00	0.12±0.00	0.08±0.001
324	0.02±0.001	10.70±0.14	0.03±0.002	0.14±0.01	0.09±0.01	0.05±0.002	5.37±0.15	0.02±0.002	42.19±1.28^{c}	0.04±0.003	40.11±1.27	0.26±0.01	0.60±0.01	0.16±0.00	0.14±0.01	0.09±0.003
332	0.02±0.002	9.64±0.64	0.03±0.01	0.12±0.02	0.09±0.03	0.05±0.02	6.35±1.09	0.02±0.002	46.95±4.21^{abc}	0.04±0.01	35.36±4.89	0.24±0.01	0.67±0.08	0.18±0.01	0.13±0.01	0.10±0.01
393	0.02±0.004	10.45±0.26	0.03±0.003	0.13±0.01	0.11±0.01	0.05±0.004	7.16±0.53	0.02±0.015	44.80±2.50^{abc}	0.04±0.002	35.75±2.95	0.26±0.02	0.76±0.04	0.16±0.00	0.15±0.01	0.10±0.001
473	0.02±0.001	8.72±0.19	0.05±0.01	0.10±0.01	0.10±0.02	0.05±0.01	6.24±0.05	0.02±0.002	52.75±1.00^{a}	0.03±0.02	30.48±1.28	0.31±0.02	0.68±0.02	0.20±0.01	0.14±0.01	010±0.002

*"*t*" means trans; Mean±SD; Means with different superscript are different ($P<0.05$).

.

Suplementary Table 6. Fatty acid composition expressed in % (g/100 g of oil) of brown sesame seeds.

Variety	Myristic acid	Palmitic acid	Palmitoleic 1 acid (ω7)	Palmitoleic 2 acid (ω7)	Margaric acid	Margaroleic acid	Stearic acid	*t*-Oleic acid	Oleic acid (ω-7+ ω-9)	*t*-linoleic 1 acid	Linoleic acid	Linolenic acid	Arachidic acid	Gadoleic acid	Behenic acid	Lignoceric acid
87	0.02±0.002	8.32±0.43	0.04±0.002	0.09±0.01	0.12±0.01	0.06±0.004	5.64±0.68	0.02±0.003	47.48±0.83^{b}	0.04±0.003	36.75±0.41	0.35±0.03	0.63±0.05	0.19±0.01	0.14±0.01	0.09±0.004
93	002±0.001	8.51±0.68	0.04±0.01	0.09±0.01	0.10±0.01	0.06±0.01	6.29±0.86	0.02±0.002	49.96±5.65^{ab}	0.04±0.01	33.43±5.92	0.34±0.05	0.65±0.08	0.19±0.01	0.13±0.01	0.09±0.01
110	0.03±0.002	7.53±0.05	0.05±0.01	0.08±0.01	0.11±0.01	0.05±0.01	6.69±0.14	0.02±0.004	51.71±0.50^{a}	0.03±0.003	32.17±0.44	0.36±0.06	0.72±0.04	0.20±0.01	0.15±0.01	0.09±0.003
167	0.02±0.003	9.00±0.35	004±0.01	0.11±0.01	0.09±0.012	0.04±0.001	6.18±0.26	0.02±0.001	48.04±1.78^{b}	0.04±0.002	35.08±2.38	0.29±0.01	0.65±0.03	0.19±0.01	0.13±0.004	0.09±0.01
168	0.02±0.002	9,.01±0.85	0.05±0.004	0.11±0.02	0.11±0.001	0.06±0.002	5.99±0.36	0.02±0.001	47.59±2.83^{b}	0.04±0.004	35.66±2.67	0.32±0.01	0.63±0.02	0.19±0.01	0.12±0.002	0.09±0.004
176	0.02±0.001	9.37±0.72	0.05±0.01	0.11±0.01	0.09±0.003	0.05±0.001	6.37±1.32	0.02±0.002	49.19±5.44^{ab}	0.03±0.01	33.28±6.13	0.29±0.05	0.69±0.12	0.19±0.01	0.14±0.02	0.11±0.02
218	0.02±0.001	9.49±0.37	0.03±0.002	0.14±0.02	0.10±0.02	0.05±0.01	5.53±0.39	0.02±0.001	42.64±3.05^{b}	0.04±0.001	40.68±3.06	0.27±0.02	0.58±0.04	0.18±0.004	0.12±0.01	0.08±0.01
228	0.02±0.003	9.85±0.39	0.05±0.01	0.16±0.02	0.10±0.02	0.06±0.01	5.18±0.37	0.02±0.002	43.82±4.77^{b}	0.04±0.01	39.35±5.28	0.37±0.05	0.59±0.04	0.18±0.01	0.13±0.004	0.09±0.01
258	0.02±0.001	9.47±0.40	0.05±0.01	0.13±0.02	0.11±0.04	0.06±0.02	5.12±0.52	0.02±0.003	42.55±2.77^{b}	0.04±0.003	41.08±3.09	0.41±0.08	0.55±0.04	0.19±0.01	0.12±0.001	0.08±0.004
265	0.02±0.001	9.45±0.23	0.04±0.01	0.12±0.01	0.11±0.01	0.06±0.01	5.67±0.12	0.02±0.003	46.06±2.18^{b}	0.04±0.003	37.10±2.34	0.32±0.05	0.58±0.02	0.19±0.02	0.12±0.004	0.08±0.01
314	0.02±0.003	9.50±0.42	0.04±0.01	0.12±0.02	0.11±0.02	0.05±0.01	6.02±0.19	0.02±0.001	46.21±1.92^{b}	0.04±0.003	36.49±1.96	0.34±0.03	0.62±0.02	0.19±0.01	0.13±0.01	0.08±0.004
427	0.02±0.001	9.37±0.48	0.04±0.003	0.13±0.004	0.14±0.03	0.08±0.02	5.72±0.22	0.02±0.001	46.85±150^{b}	0.04±0.002	36.25±0.82	0.29±0.01	0.62±0.03	0.19±0.004	0.13±0.01	0.09±0.01
483	0.02±0.003	8.48±0.07	0.05±0.01	0.11±0.002	0.11±0.004	0.06±0.002	6.29±0.45	0.02±0.001	50.52±3.63^{a}	0.04±0.01	33.01±0.27	0.27±0.03	0.64±0.03	0.19±0.004	0.12±0.001	0.08±0.003
484	0.02±0.002	8.92±0.12	0.04±0.003	0.11±0.01	0.12±0.02	0.06±0.01	5.66±0.27	0.02±0.002	47.44±2.59^{b}	0.04±0.003	36.32±2.98	0.27±0.02	0.58±0.01	0.19±0.01	0.11±0.01	0.08±0.000

*"t" means trans; Mean±SD; Means with different superscript are different ($P<0.05$).

Supplementary Table 7. Fatty acid composition expressed in % (g/100 g of oil) of black sesame seeds.

Variety	Myristic acid	Palmitic acid	Palmitoleic 1 acid (ω7)	Palmitoleic 2 acid (ω7)	Margaric acid	Margaroleic acid	Stearic acid	*t*-oleic acid	Oleic acid (ω-7+ ω-9)	*t*-linoleic 1 acid	Linoleic acid	Linolenic acid	Arachidic acid	Gadoleic acid	Behenic acid	Lignoceric acid
141	0.02±0.001	8.94±0.55	0.03±0.001	0.11±0.01	0.10±0.004	0.05±0.002	5.72±0.44	0.02±0.001	47.67±1.03^{abcd}	0.04±0.004	35.92±1.28^{abc}	0.26±0.01	0.65±0.06	0.20±0.01	0.15±0.01	0.11±0.01
152	0.02±0.001	9.55±0.18	0.04±0.002	0.13±0.01	0.11±0.01	0.06±0.01	5.21±0.37	0.02±0.002	45.22±2.13^{abcd}	0.04±0.001	38.35±2.55^{abc}	0.29±0.04	0.58±0.03	0.18±0.002	0.12±0.004	0.08±0.01
153	0.02±0.001	9.04±0.63	0.04±0.01	0.13±0.02	0.10±0.01	0.05±0.004	6.08±0.66	0.02±0.004	48.05±3.64^{ab}	0.03±0.001	35.06±3.73^{abc}	0.26±0.01	0.68±0.07	0.18±0.01	0.15±0.01	0.10±0.01
157	0.02±0.001	8.54±0.49	0.05±0.01	0.10±0.01	0.11±0.02	0.06±0.01	6.15±0.63	0.02±0.01	51.05±3.40^{a}	0.03±0.01	32.57±3.51^{c}	0.28±0.03	0.62±0.05	0.19±0.004	0.13±0.01	0.08±0.001
161	0.02±0.002	8.55±0.52	0.03±0.001	0.10±0.002	0.10±0.02	0.05±0.01	6.66±0.18	0.02±0.03	43.78±2.11^{abcd}	0.04±0.003	39.26±1.91^{abc}	0.29±0.02	0.69±0.01	0.19±0.001	0.14±0.002	0.09±0.004
170	0.03±0.002	10.82±0.91	0.04±0.001	0.15±0.02	0.10±0.001	0.05±0.01	5.34±0.29	0.02±0.01	43.07±3.60^{abcd}	0.04±0.002	39.03±2.80^{abc}	0.35±0.06	0.59±0.02	0.17±0.01	0.12±0.01	0.08±0.001
216	0.02±0.004	9.89±0.50	0.03±0.01	0.14±0.001	0.12±0.03	0.07±0.02	5.23±0.41	0.02±0.003	40.93±2.59^{cd}	0.04±0.004	42.14±2.09^{a}	0.31±0.05	0.62±0.06	0.19±0.02	0.14±0.02	0.09±0.01
271	0.02±0.003	10.24±0.49	0.04±0.02	0.14±0.02	0.11±0.01	0.06±0.004	4.96±0.08	0.02±0.001	42.29±1.22^{bcd}	0.04±0.01	40.80±0.57^{ab}	0.33±0.08	0.56±0.03	0.18±0.01	0.12±0.01	0.09±0.001
282	0.02±0.002	9.86±0.63	0.04±0.01	0.14±0.02	0.11±0.01	0.05±0.01	6.00±0.87	0.02±0.004	48.71±4.33^{ab}	0.03±0.01	33.68±4.81^{bc}	0.26±0.04	0.66±0.10	0.18±0.001	0.14±0.02	0.10±0.01
284	0.02±0.001	9.24±0.24	0.03±0.003	0.13±0.001	0.08±0.002	0.04±0.004	5.41±0.15	0.02±0.02	45.41±0.92^{abcd}	0.04±0.002	38.33±0.85^{abc}	0.27±0.01	0.60±0.01	0.18±0.004	0.13±0.01	0.09±0.002
302	0.02±0.001	9.62±0.60	0.03±0.002	0.13±0.01	0.10±0.01	0.05±0.002	5.35±0.18	0.02±0.01	43.64±1.43^{abcd}	0.04±0.003	39.77±1.29^{abc}	0.27±0.03	0.57±0.01	0.19±0.001	0.12±0.01	0.08±0.001
329	0.02±0.001	10.40±0.34	0.03±0.003	0.15±0.01	0.10±0.02	0.05±0.01	5.33±0.19	0.02±0.004	40.35±2.34^{d}	0.05±0.01	42.27±2.24^{a}	0.26±0.01	0.59±0.02	0.16±0.003	0.12±0.004	0.09±0.003
358	0.02±0.001	10.10±0.15	0.04±0.01	0.13±0.01	0.11±0.01	0.05±0.01	6.48±0.37	0.02±0.003	42.21±4.04^{bcd}	0.04±0.004	39.28±4.06^{abc}	0.42±0.12	0.69±0.05	0.18±0.002	0.14±0.01	0.10±0.002
403	0.02±0.004	9.22±0.24	0.04±0.02	0.12±0.003	0.10±0.01	0.05±0.004	5.09±0.30	0.02±0.004	42.70±3.54^{bcd}	0.04±0.002	41.25±3.73^{ab}	0.35±0.13	0.59±0.02	0.19±0.004	0.13±0.01	0.09±0.002
463	0.02±0.003	9.13±0.45	0.04±0.001	0.11±0.001	0.11±0.01	0.06±0.01	5.11±0.32	0.02±0.001	43.70±0.33^{abcd}	0.04±0.003	40.37±0.52^{ab}	0.30±0.01	0.58±0.02	0.20±0.01	0.13±0.01	0.09±0.004
508	0.02±0.001	8.62±1.00	0.04±0.01	0.11±0.02	0.10±0.01	0.05±0.01	6.67±0.50	0.02±0.004	49.02±0.66^{ab}	0.03±0.004	33.98±0.13^{bc}	0.22±0.01	0.68±0.04	0.20±0.01	0.13±0.01	0.10±0.01

*"t" means trans; Mean±SD; Means with different superscript are different ($P<0.05$).

Supplementary Table 8. Fatty acid composition expressed in % (g/100 g of oil) of selected sesame seeds roasted at 180°C and 250°C .

White	Myristic acid	Palmitic acid	Palmitoleic 1 acid (ω7)	Palmitoleic 2 acid (ω7)	Margaric acid	Margaroleic acid	Stearic acid	*t*-oleic acid	Oleic acid (ω-7+ ω-9)	*t*-linoleic 1 acid	t-linoleic 2 acid	Linoleic acid	*t*-linolenic acid	Linolenic acid	Arachidic acid	Gadoleic acid	Behenic acid	Lignoceric acid
121 (180°C)	0.02±0.001	9.40±0.07	0.03±0.01	0.11±0.01	0.11±0.01	0.06±0.002	5.56±0.34	0.11±0.03	45.77±3.86	0.10±0.003	0.05±0.01	37.15±4.29	n.d.	0.29±0.01	0.64±0.04	0.20±0.01	0.14±0.002	0.10±0.01
121 (250°C)	0.02±0.002	9.40±0.17	0.03±0.01	0.11±0.02	0.11±0.004	0.05±0.003	5.89±0.39	1.11±0.12	45.30±3.37	0.90±0.09	0.83±0.08	34.88±3.92	0.01±0.002	0.24±0.01	0.66±0.04	0.22±0.01	0.14±0.002	0.10±0.01
322 (180°C)	0.02±0.002	10.01±0.27	0.03±0.003	0.13±0.01	0.09±0.01	0.05±0.004	5.43±0.54	0.12±0.02	45.34±1.53	0.11±0.01	0.06±0.01	37.44±2.32	n.d.	0.23±0.01	0.57±0.04	0.17±0.004	0.12±0.002	0.08±0.01
322 (250°C)	0.02±0.002	10.03±0.22	0.03±0.003	0.13±0.01	0.09±0.01	0.05±0.01	5.46±0.46	1.12±0.06	44.01±1.79	0.95±0.06	0.88±0.05	35.99±2.35	0.01±0.004	0.19±0.01	0.58±0.04	0.26±0.01	0.12±0.003	0.08±0.01
**Black**																		
286/1 (180°C)	0.02±0.001	9.84±0.42	0.05±0.01	0.14±0.01	0.11±0.01	0.06±0.003	5.75±0.51	0.15±0.01	47.92±2.56	0.12±0.01	0.07±0.01	34.65±3.03	n.d.	0.28±0.01	0.45±0.38	0.18±0.01	0.13±0.02	0.10±0.01
286/1 (250°C)	0.02±0.002	9.82±0.55	0.05±0.01	0.13±0.02	0.11±0.01	0.05±0.01	6.07±0.70	1.29±0.11	48.02±3.57	0.88±0.11	0.82±0.10	31.40±3.76	0.01±0.002	0.23±0.01	0.66±0.08	0.19±0.01	0.13±0.02	0.10±0.01
333 (180°C)	0.02±0.001	10.29±0.16	0.03±0.002	0.15±0.01	0.09±0.02	0.05±0.01	5.34±0.09	0.10±0.02	40.59±1.68	0.11±0.02	0.06±0.02	41.94±1.69	n.d.	0.27±0.01	0.59±0.02	0.16±0.003	0.12±0.01	0.09±0.02
333 (250°C)	0.02±0.002	10.38±0.08	0.03±0.003	0.15±0.004	0.10±0.02	0.05±0.01	5.95±0.61	0.99±0.13	40.32±1.36	0.99±0.04	0.91±0.05	39.14±1.54	0.01±0.003	0.22±0.01	0.60±0.02	0.26±0.01	0.13±0.003	0.09±0.02

"*t*" means trans, Mean±SD
